# mTORC1 Mediates Biphasic Mechano‐Response to Orchestrate Adhesion‐Dependent Cell Growth and Anoikis Resistance

**DOI:** 10.1002/advs.202307206

**Published:** 2023-12-02

**Authors:** Chunlei Zhang, Yuan Wang, Zifeng Zhen, Jiayi Li, Jing Su, Congying Wu

**Affiliations:** ^1^ Institute of Systems Biomedicine School of Basic Medical Sciences Peking University Health Science Center Beijing 100191 China; ^2^ International Cancer Institute Beijing Key Laboratory of Tumor Systems Biology Peking University Health Science Center Beijing 100191 China; ^3^ Pathology Department Peking University Third Hospital Beijing 100191 China

**Keywords:** autophagy, cell adhesion, mechano‐transduction, mTORC1

## Abstract

Cells constantly sense and respond to not only biochemical but also biomechanical changes in their microenvironment, demanding for dynamic metabolic adaptation. ECM stiffening is a hallmark of cancer aggressiveness, while survival under substrate detachment also associates with poor prognosis. Mechanisms underlying this, non‐linear mechano‐response of tumor cells may reveal potential double‐hit targets for cancers. Here, an integrin‐GSK3β‐FTO‐mTOR axis is reported, that can integrate stiffness sensing to ensure both the growth advantage endowed by rigid substrate and cell death resistance under matrix detachment. It is demonstrated that substrate stiffening can activate mTORC1 and elevate mTOR level through integrins and GSK3β‐FTO mediated mRNA m^6^A modification, promoting anabolic metabolism. Inhibition of this axis upon ECM detachment enhances autophagy, which in turn conveys resilience of tumor cells to anoikis, as it is demonstrated in human breast ductal carcinoma in situ (DCIS) and mice malignant ascites. Collectively, these results highlight the biphasic mechano‐regulation of cellular metabolism, with implications in tumor growth under stiffened conditions such as fibrosis, as well as in anoikis‐resistance during cancer metastasis.

## Graphical abstract

Mechanical force participates in various biological processes that require metabolic rewiring. As the major regulator of anabolism, mTORC1 has been the highlight of research in cell growth and tumor survival. An integrin‐GSK3β‐FTO‐mTOR axis is demonstrated to mediate the biphasic roles of mTORC1 in both tumor cell proliferation under high adhesion conditions/on stiff substrates and anoikis resistance upon substrate detachment.

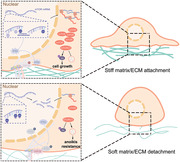

## 1 Introduction

The physical properties of the ECM are sensed by cells via integrin‐conjugated focal adhesions (FAs), influencing cell migration, differentiation, and survial.^[^ ^1^, ^2^, ^3^, ^4^, ^5^ ^]^ Due to that tissue fibrosis and ECM stiffening associate with poor tumor prognosis,^[^ ^6^, ^7^ ^]^ changing ECM rigidity via collagen depletion or lysyl oxidase (LOX) inhibition, or pharmaceutically altering cellular mechano‐transduction have been promising options for cancer therapy.^[^ ^8^, ^9^, ^10^ ^]^ However, tumor cells also exhibit resistance to anoikis‐the ECM detachment‐induced cell death. How tumor cells manage to gain growth advantage on stiff substrate while resist cell death upon matrix detachment remain enigmatic.

Cell growth and proliferation are intimately linked to cellular anabolism. Anoikis‐resistance also occurs concomitantly with metabolic adaptation^[^ ^11^ ^]^ and can be hindered by autophagy. The mechanistic target of rapamycin complex 1 (mTORC1) is a central pillar in metabolic regulation in response to both intra‐ and extra‐cellular signals.^[^ ^12^, ^13^, ^14^, ^15^, ^16^, ^17^, ^18^ ^]^ Its activation associates with enhanced protein translation^[^ ^19^, ^20^ ^]^ and decreased autophagy.^[^ ^21^, ^22^ ^]^ mTORC1 is recruited to the lysosomal surface^[^ ^15^ ^]^ to release its inhibition. Recent studies indicated that lysosomal mTORC1 could be transported to and activated around FAs^[^ ^23^ ^]^—the integrin‐based mechano‐transducing hub that links ECM with the cell interior.^[^ ^24^, ^25^ ^]^ However, there still lacks evidence of whether physical properties of the matrix can be signaled to the mTORC1 pathway.

Here we discovered the phenomenon and the underlying mechanism of mechano‐regulation of the mTORC1 pathway on stiff matrix or upon substrate detachment. Increased substrate rigidity and cellular tension enhanced mTOR activity and abundance via integrins, GSK3β, and mRNA m^6^A modification involving the “eraser” protein FTO, favoring cell growth. Meanwhile, the same set of machineries were employed by tumor cells under ECM detachment where reduced mTORC1 activity and enhanced autophagy were observed in vitro, as well as in human breast ductal carcinoma in situ (DCIS) and mice malignant ascites, accompanying anoikis resistance. Our findings suggest a biphasic mechano‐regulation of cellular metabolism harnessed by tumor cells to orchestrate cell growth on stiff matrix and anoikis resistance upon substrate detachment. Targeting the integrins‐GSK3β‐FTO‐mTOR‐axis may reveal double‐hit strategies for cancers.

## 2 Results

### 2.1 mTORC1 Activation Mediates Cell Growth Increase Under Stiff Culture Conditions

**Figure 1:**
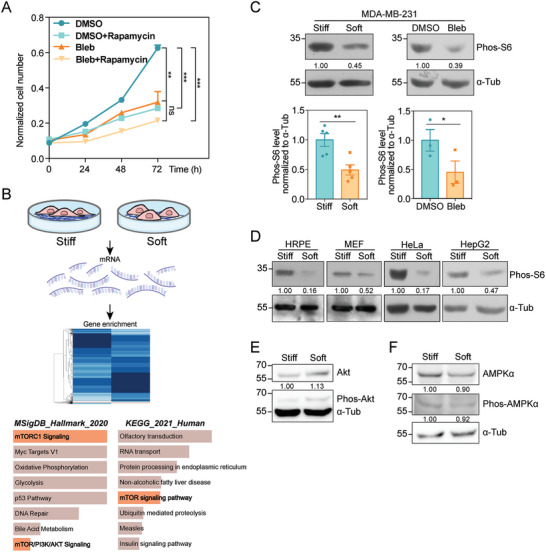
Substrate stiffness activates mTORC1 pathway to promote cell growth. A) Cell growth curve determined by CCK‐8 assay at 0, 24, 48, and 72 h. Cell number was measured in DMSO and Bleb groups with or without 40 nM Rapamycin treatment (*N*_DMSO_ = *N*_DMSO+Rapamycin_ = *N*_Bleb_ = *N*_Bleb+Rapamycin_ = 3 independent samples, error bar: mean with SEM, ns = 0.8846, ***p_DMSO versus DMSO+Rapamycin_ = 0.0008, ***p_DMSO versus Bleb_ = 0.0005, **p_DMSO versus Bleb+Rapamycin_ = 0.0015 by one‐way *ANOVA* for multiple comparison). B) Schematic diagram of RNA‐Seq assay followed by gene enrichment of MDA‐MB‐231 cells plated on stiff or soft substrates. The graphs display the 8 most significantly enriched gene sets based on MSigDB or KEGG database, analyzed with Enrichr and ranked according to combined score. Gene sets related to mTOR signaling were highlighted in light red. C) Top: western blot showing the Phos‐S6 levels in MDA‐MB‐231 cells plated on stiff or soft substrates (left), treated with DMSO or Bleb (right). α‐tubulin is used as loading control. Bottom: quantification of Phos‐S6 levels normalized to α‐tubulin accordingly (Error bar: mean with SEM, *p_stiff versus soft_ = 0.0133 and *p_DMSO versus Bleb_ = 0.0156 by paired Student's *t*‐test). D) Western blot showing the Phos‐S6 levels in HRPE, MEF, HeLa, HepG2 cells plated on stiff or soft substrates. α‐tubulin is used as loading control. E) Western blot showing Phos‐Akt and total Akt levels in MDA‐MB‐231 cells plated on stiff or soft substrates. α‐tubulin is used as loading control. F) Western blot showing Phos‐AMPKα and total AMPKα levels in MDA‐MB‐231 cells plated on stiff or soft substrates. α‐tubulin is used as loading control.

Cell proliferation, as measured by cell number increase, was markedly enhanced on stiff culture dishes (at gigapascals level, ∼GPa)^[^ ^26^, ^27^ ^]^ compared to soft poly‐acrylamide gels (about 4 kilopascals, ~4 kPa), or upon vehicle treatment compared to contractility inhibition by the myosin II inhibitor blebbistatin (Figure S1A, Supporting Information, and **Figure** **1**A). To explore the major players involved in the growth advantages endowed by stiff substrate, we enriched signature genes upregulated on stiff substrate from RNA‐Seq (Figure 1B). Notably, consistent with previous findings that metabolic control underlies cell growth adaptation to extracellular stimuli,^[^ ^28^, ^29^, ^30^ ^]^ the mTORC1 signaling pathway was enriched in both MsigDB and KEGG databases. To verify the role of mTORC1 in cell proliferation regulation by substrate stiffness, we used rapamycin to inhibit mTORC1 and compared cell proliferation under a vehicle or blebbistatin treatment. In line with previous studies,^[^ ^31^, ^32^ ^]^ rapamycin decreased cell growth. Moreover, the proliferation advantage in vehicle treatment group was also diminished under mTORC1 inhibition (Figure 1A). To exclude the possibility that rapamycin decreased cell proliferation to the extent that further growth reduction upon inhibiting cell contractility would be negligible, we tested whether rapamycin can abrogate the effect of myosin light chain (MLC) overexpression (Figure S1B, Supporting Information) to rescue cell proliferation decrease upon contractility inhibition. In contrast to the moderate but significant growth rate increase in overexpression cells, rapamycin abolished any noticeable growth changes between these groups (Figure S1C, Supporting Information). These results suggest that mTORC1 is involved in cell growth modulation downstream of matrix rigidity and cellular contraction.

Next, we asked whether stiff matrix or enhanced cell contractility could activate mTORC1 activity by probing the phosphorylation of S6.^[^ ^33^, ^34^ ^]^ Indeed, we detected a significant increase in phos‐S6 level under stiff culture condition compared to soft matrix, whereas no obvious changes were observed in total S6 level (Figure 1C and Figure S1D, Supporting Information). Moreover, both blebbistatin or Y27632‐that inhibits ROCK, recapitulated the decreased mTORC1 activity in soft‐cultured cells (Figure 1C and Figure S1E, Supporting Information). Reciprocally, when cells on soft gels were treated with Rho activator II to enhance contractility, the mTORC1 activity was restored to the similar level in stiff‐cultured cells (Figure S1F, Supporting Information). We then extended the above findings in a variety of cell lines other than the MDA‐MB‐231 cells, including normal epithelial and other transformed cancer cells (Figure 1D). Considering the fact that mTORC1 shares multiple subunits including the core protein mTOR with the mTORC2, we also examined whether matrix stiffness also affected mTORC2 activation. However, no obvious changes in Akt phosphorylation‐which reveals mTORC2 activity‐were identified in cells on stiff versus soft substrate (Figure 1E), suggesting the specificity of mTORC1 activation under stiff conditions. These observations demonstrate that mTORC1 activation may be a widespread response to substrate stiffening or cytoskeletal contraction.

mTORC1 and AMPK play Yin‐Yang roles during cell growth and survival by propelling anabolic and catabolic processes respectively.^[^ ^35^ ^]^ Our studies validated the conclusion in a previous report which suggested that soft matrix may decrease AMPK level in stromal cells under the serum‐starved condition,^[^ ^36^ ^]^ however, we failed to detect the changes of S6 phosphorylation under different substrate stiffness (Figure S1G, Supporting Information). In contrast, we were unable to detect significant changes in either AMPKα protein abundance or AMPKα1/α2 phosphorylation level between cells on stiff versus soft substrate under normal serum condition (Figure S1F, Supporting Information). These observations indicate an intriguing rewiring of the mechano‐metabolic regulation under different nutrient states. To delineate a more general cellular response to mechanical loading imposed by substrate stiffness, we thus focused on the mTORC1 pathway.

### 2.2 Focal Adhesions Mediate mTORC1 Activation by Matrix Rigidity

**Figure 2:**
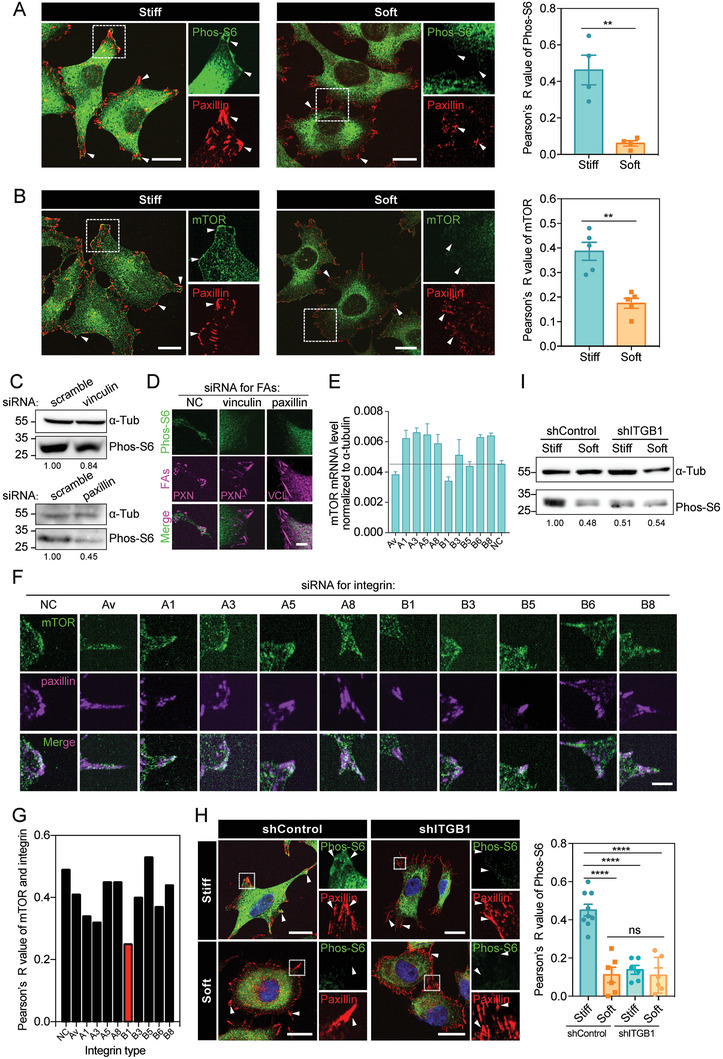
mTORC1 activation on stiff matrix is regulated by integrin‐based FAs A) Left: representative immunofluorescence images stained with Phos‐S6 (green) and paxillin (red) antibodies in MDA‐MB‐231 cells plated on stiff or soft substrates. Co‐localization of Phos‐S6 and paxillin is shown in enlarged boxes by white arrow heads. Scale bar, 20 µm. Right: Pearson's R value represents the overlap coefficient of Phos‐S6 and paxillin (*N*_stiff_ = 3, N_soft_ = 4, error bar: mean with SEM, **p = 0.0028 by unpaired Student's *t*‐test). B) Left: representative immunofluorescence images stained with mTOR (green) and paxillin (red) antibodies in MDA‐MB‐231 cells plated on stiff or soft substrates. Co‐localization of mTOR and paxillin is shown in enlarged boxes by white arrow heads. Scale bar, 20 µm. Right: Pearson's *R*‐value represents the overlap coefficient of mTOR and paxillin (*N*_stiff_ = 5, *N*_soft_ = 5, error bar: mean with SEM, **p = 0.0011 by unpaired Student's *t*‐test). C) Top: western blot showing the Phos‐S6 levels in vinculin‐silenced MDA‐MB‐231 cells. Bottom: western blot showing the Phos‐S6 levels in paxillin‐silenced MDA‐MB‐231 cells. α‐tubulin is used as loading control. D) Representative immunofluorescence images stained with Phos‐S6 (green), vinculin (magenta) or paxillin (magenta) antibodies in siNC, si‐vinculin or si‐paxillin cells. Scale bar, 5 µm. E) Bar chart shows the normalized mTOR mRNA level in siNC and integrin AV/A1/A3/A5/A8/B1/B3/B5/B6/B8 gene‐silenced MDA‐MB‐231 cells. The black line means the mTOR mRNA level in siNC cells. Error bar: mean with SEM. F) Representative immunofluorescence images stained with Phos‐S6 (green) and paxillin (magenta) antibodies in siNC and integrin AV/A1/A3/A5/A8/B1/B3/B5/B6/B8 gene‐silenced MDA‐MB‐231 cells. Scale bar, 5 µm. G) Pearson's *R*‐value represents the overlap coefficient of mTOR and paxillin in different integrin siRNA‐treated cells. The lowest integrin type is shown in red. H) Left: representative immunofluorescence images stained with Phos‐S6 (green) and paxillin (red) antibodies in shControl and shITGB1 cells on stiff or soft substrates. Co‐localization of Phos‐S6 and paxillin is shown in enlarged boxes by white arrow heads. Scale bar, 20 µm. Right: Pearson's R‐value represents the overlap coefficient of Phos‐S6 and paxillin (shControl: *N*_stiff_ = 9, N_soft_ = 6; shITGB1: *N*_stiff_ = 6, *N*_soft_ = 6; error bar: mean with SEM, ****p < 0.0001 by one‐way ANOVA for multiple comparison). I) Western blot showing Phos‐S6 levels in shControl and shITGB1 cells on stiff or soft substrates. α‐tubulin is used as a loading control.

Integrin‐based focal adhesions (FAs) sense the physical properties of ECM and convey mechanical cues into cells.^[^ ^37^, ^38^ ^]^ Interestingly, it has been shown recently that lysosome‐bound mTORC1, suggestive of its active form, is translocated to the FAs for its full activation downstream of extracellular growth‐promoting stimuli.^[^ ^39^ ^]^ This prompted us to examine whether FAs participate in substrate stiffness‐induced mTORC1 activation. First, using immunofluorescent staining, we detected strong co‐localization of mTOR with FA marker proteins paxillin or vinculin, on stiff but not soft matrix (**Figure** **2**A and Figure S2A, Supporting Information). Similar scenario of stiff matrix‐specific FA localization also occurred with phos‐S6, which indicated mTORC1 activation (Figure 2B and Figure S2B, Supporting Information). Next, when we perturbed cell contractility in stiff‐cultured cells using blebbistatin, the specific FA localization of mTOR and phos‐S6 disappeared, as evidenced with both confocal and super‐resolution STED imaging (Figure S2C–E, Supporting Information). Third, we knocked down vinculin or paxillin to investigate whether FA disruption would diminish mTORC1 activity under stiff‐culture condition (Figure S2F, Supporting Information). Indeed, we detected loss of FA localization of phos‐S6 and decreased phos‐S6 level (Figure 2C,D). Cumulatively, these data suggest that FAs play a critical role in stiff matrix‐induced mTORC1 activation.

To further dissect the mechanism underlying the mechano‐activation of mTORC1, we focused on integrins, which linked ECM with cytoskeletons and transduced mechanical cues across the plasma membrane.^[^ ^24^ ^]^ To this end, we performed siRNA screen library targeting various integrins and validated the good knockdown efficiency of these siRNAs (Figure S2G, Supporting Information). Next, we examined the effect of knockdown of different integrins on mTORC1 regulation. We found that integrin αV or β1 knockdown decreased mTOR mRNA and protein levels, while knockdown of β1, α5, or α8 inhibited mTORC1 activity, both mTOR protein level and mTORC1 activity decreased only upon knocking down integrin β1 (Figure 2E and Figure S2H, Supporting Information). Based on these results, we finally chose integrin β1 for further study. We thus set to investigate whether integrin β1 participated in mTORC1 regulation. Interestingly, integrin β1 knockdown hampered the co‐localization between mTOR/phos‐S6 and paxillin on stiff substrate, and decreased mTORC1 activity as assayed by the phos‐S6 level (Figure 2F–I and Figure S2I, Supporting Information). These results reveal an important role of integrin β1 in stiffness‐mediated mTORC1 activation.

### 2.3 Soft Matrix Decreases mTOR Protein Abundance by Regulating m^6^A RNA Methylation

**Figure 3:**
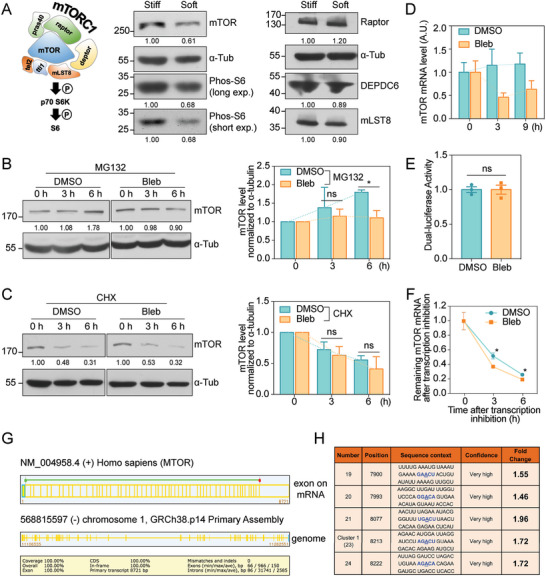
Substrate rigidity mediates mTOR abundance and m^6^A level. A) Left: schematic diagram of the signaling cascade from mTORC1 complex to S6. Middle and Right: western blot showing the levels of mTOR, Phos‐S6 (long exposure), Phos‐S6 (short exposure), Raptor, DEPDC6, and mLST8 in MDA‐MB‐231 cells cultured on stiff or soft substrates. α‐tubulin is used as loading control. B) Left: Western blot showing the mTOR protein synthesis process in MDA‐MB‐231 cells treated with DMSO or Bleb, each exposed to MG132 for 0, 3, 6 h. α‐tubulin is used as loading control. Right: quantification of the mTOR levels normalized to α‐tubulin (*N* = 3 independent experiments, error bar: mean with SEM, ns = 0.9227, *p = 0.0100 by paired Student's *t*‐test). C) Left: Western blot showing the mTOR protein degradation in MDA‐MB‐231 cells treated with DMSO or Bleb, each exposed to CHX for 0, 3, and 6 h. α‐tubulin is used as loading control. Right: quantification of the mTOR levels normalized to α‐tubulin (*N* = 3 independent experiments, error bar: mean with SEM, ns = 0.4047, ns = 0.6403 by paired Student's *t*‐test). D) QPCR assay showing the mTOR mRNA levels in MDA‐MB‐231 cells treated with DMSO or Bleb at 0, 3, and 9 h (Error bar: mean with 95% Cl). E) Dual‐luciferase reporter assay showing transcriptional activity of mTOR promoter in HEK293T cells treated with DMSO or Bleb (Error bar: mean with SEM, ns > 0.9999 by unpaired Student's *t*‐test). F) The remaining mTOR mRNA levels at 0, 3, 6 h after transcription inhibition using Actinomycin D in DMSO and Bleb groups (Error bar: mean with SEM, *p_3 h_ = 0.0259, *p_6 h_ = 0.0175 by unpaired Student's *t*‐test). Prediction by SRAMP showing five very high confidence m^6^A modification loci of mTOR mRNA. G) The schematic diagram shows the 58 exons information of the mTOR transcript predicted by the NCBI‐Splign website (https://www.ncbi.nlm.nih.gov/sutils/splign/) and the corresponding genomic region. The details of the transcript are shown at the bottom. H) Table summary of the five very confidence m^6^A sites on mTOR mRNA, containing the position, sequence, and fold change after blebbistatin treatment. Fold change is analyzed from MeRIP‐qPCR assay.

Aside from the lower mTORC1 activation on soft matrix (Figure 1), we wondered whether core components of the mTORC1 were perturbed under soft culture condition or upon decreased actomyosin contraction. Interestingly, we found that cells on soft substrate or knocked down of vinculin exhibited markedly decreased mTOR protein level without changes in Raptor, mLST8, or DEPDC6 abundance (**Figure** **3**A and Figure S3A, Supporting Information). Reciprocally, when cells were treated with Rho activator II, the mTOR abundance on soft gels was restored (Figure S3B, Supporting Information). Lowered protein abundance can result from restricted protein synthesis and/or enhanced degradation. On one hand, to examine whether mTOR protein synthesis is hampered upon reduced cellular contraction, we inhibited proteasomal degradation by MG132 and monitored mTOR protein level in cells treated with blebbistatin or DMSO as vehicle control. Consistent with its role in reducing the degradation of ubiquitin‐conjugated proteins, MG132 led to an accumulation of mTOR protein in control group. In contrast, this increase was blunted in blebbistatin‐treated cells (Figure 3B), suggesting that mTOR protein synthesis may be hampered under low‐force conditions, resulting in decreased total mTOR. On the other hand, we inhibited translation elongation by cycloheximide (CHX), and probed mTOR protein level changes in control versus blebbistatin‐treated cells. However, no obvious difference in mTOR degradation was observed between these two groups (Figure 3C). Taken together, the decrease in mTOR level under soft matrix or lowered contraction force was due to suppressed protein synthesis rather than enhanced degradation.

Having observed that cells on soft substrate exhibited hampered mTOR synthesis, we then interrogated the upstream regulations, such as translation, transcription, and mRNA modification, that may lead to this phenomenon. To examine whether protein translation play a key role here, we used polysome profiling assay to evaluate mTOR mRNA level in translation‐active polysomes^[^ ^40^ ^]^ of cells under different contractility conditions. However, we failed to observe a significant difference of actively‐translated mTOR transcript levels in blebbistatin versus DMSO groups (Figure S3C, Supporting Information). Notably, mTOR mRNA was reduced upon blebbistatin treatment, as probed by quantitative PCR (qPCR) (Figure 3D). Decreased mRNA level may result from dampened transcription capacity or disturbed RNA stability. When we probed mTOR mRNA transcription capacity using the dual‐luciferase reporter system containing the *Renilla* luciferase vector and mTOR promoter‐firefly luciferase vector, we failed to observe significant difference of luciferase activity between DMSO and blebbistatin treated cells (Figure 3E and Figure S3D, Supporting Information). In contrast, when we blocked RNA synthesis using actinomycin D and monitored mRNA stability, we observed significantly faster degradation of mTOR mRNA upon blebbistatin treatment (Figure 3F). These results suggest that the lowered transcripts level under inhibited contractility condition may be the consequence of decreased mTOR mRNA stability.

As the most abundant and prevalent modification on mammalian mRNAs^[^ ^41^, ^42^ ^]^‐methylation at the N6 position of adenosine (m^6^A) has been evidenced to regulate cell metabolism and survival by affecting mRNA stability and translation, in particular when the methylation takes place at the key metabolic transcripts.^[^ ^41^, ^43^ ^]^ Since mTOR transcript contains several long exons (Figure 3G), which can be regulated by m^6^A machinery,^[^ ^44^, ^45^, ^46^, ^47^ ^]^ we asked whether m^6^A modification might mediate mechano‐regulation of mTOR mRNA and protein abundance. The online software SRAMP^[^ ^48^ ^]^ predicted five very high confident m^6^A modification loci located at the 3′UTR of mTOR mRNA (Figure 3H and Figure S3E, Supporting Information). Based on the prediction, we analyzed m^6^A level on these potentially modified sites using gene‐specific m^6^A RNA‐immunoprecipitation‐qPCR (MeRIP‐qPCR). Noticeably, all five sites exhibited elevated m^6^A modification upon blebbistatin treatment (Figure S3F, Supporting Information). Taken together, our data argue that rigidity sensitive m^6^A changes may mediate alterations in mTOR protein abundance under different stiffness or cell contractility conditions.

### 2.4 FTO Phosphorylation Links Rigidity Sensing with mTOR m^6^A Modification

**Figure 4:**
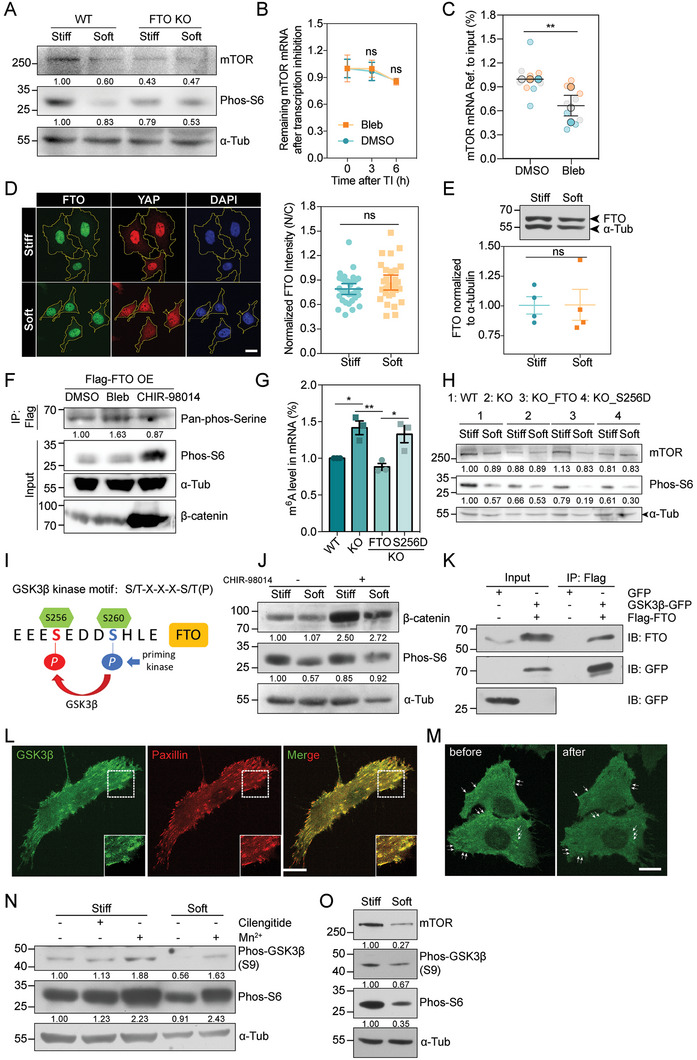
GSK3β plays a crucial role in phosphorylating FTO through interaction with integrins. A) Western blot showing the mTOR and Phos‐S6 levels in WT and FTO KO cells on stiff or soft substrates. α‐tubulin is used as loading control. B) The remaining mTOR mRNA levels at 0, 3, 6 h after transcription inhibition (TI) using Actinomycin D in FTO KO cells treated with DMSO or Bleb (Error bar: mean with SEM, ns_3 h_ = 0.8436, ns_6 h_ = 0.7978 by unpaired Student's *t*‐test). C) RIP‐qPCR assay showing the relative levels of mTOR mRNA enriched by FTO in DMSO and Bleb groups (*N*_DMSO_ = *N*_Bleb_ = 9, error bar: mean with SEM, **p = 0.0091 by paired Student's *t*‐test). D) Left: representative immunofluorescence images stained with FTO (green) and YAP (red) antibodies in MDA‐MB‐231 cells cultured on stiff or soft substrates. Scale bar, 20 µm. Right: FTO intensity measured by the ratio of nucleus/cytoplasm (*N*_stiff_ = 31, *N*_soft_ = 29, error bar: mean with 95% CI, ns = 0.1548 by unpaired Student's *t*‐test). E) Top: western blot showing the FTO levels in MDA‐MB‐231 cells cultured on stiff or soft substrates. α‐tubulin is used as loading control. Bottom: quantification of the FTO levels normalized to α‐tubulin (*N*_stiff_ = 4, *N*_soft_ = 4, error bar: mean with SEM, ns = 0.9703 by paired Student's *t*‐test). F) Co‐immunoprecipitation assay showing the pan‐phosphorylated serine level of FTO, Phos‐S6 and β‐catenin levels in DMSO, Bleb and CHIR‐98014 treated groups. Flag‐FTO was over‐expressed in HEK293T cells. α‐tubulin is used as loading control. G) The total m^6^A ratios on mRNA in WT, KO, KO_FTO, KO_S256D cells (*N*_WT_ = *N*_KO_ = *N*_KO_FTO_ = *N*_KO_S256D_ = 3 independent experiments, error bar: mean with SEM, *p_WT versus KO_ = 0.0222, ns_WT versus KO_FTO_ = 0.7161, ns_WT versus KO_S256D_ = 0.0673, **p_KO versus KO_FTO_ = 0.0055, ns_KO versus KO_S256D_ = 0.8546, *p_KO_FTO versus KO_S256D_ = 0.0153 by one‐way *ANOVA* for multiple comparison). H) Western blot showing the mTOR and Phos‐S6 levels in WT, KO, KO_FTO, KO_S256D cells cultured on stiff or soft substrates. α‐tubulin is used as loading control. I) The consensus GSK3β phosphorylation motif in human FTO. The numbering S256 and S260 indicate amino acid position within FTO protein, S260 was primed by various kinases. J) Western blot showing the β‐catenin and Phos‐S6 levels in MDA‐MB‐231 cells cultured on stiff or soft substrates with or without CHIR‐98014 treatment. α‐tubulin is used as loading control. K) Co‐immunoprecipitation assay showing the interaction between GSK3β‐GFP and Flag‐FTO. L) Representative immunofluorescence images stained with GSK3β (green) and paxillin (red) antibodies in MDA‐MB‐231 cells. Dashed boxes are zoomed in on the lower right. Scale bar, 20 µm. M) Representative images of with GSK3β‐AcGFP in MDA‐MB‐231 cells before and after Mn^2+^ treatment. The white arrows indicate localization of GSK3β at the bottom of the cell. Scale bar, 20 µm. N) Western blot showing the Phos‐GSK3β (S9) and Phos‐S6 levels in MDA‐MB‐231 cells on stiff and soft substrates with or without Mn^2+^ or Cilengitide treatment. α‐tubulin is used as loading control. O) Western blot showing the mTOR, Phos‐GSK3β (S9) and Phos‐S6 levels in MDA‐MB‐231 cells cultured on stiff or soft substrates. α‐tubulin is used as loading control.

The level of mRNA m^6^A is regulated by m^6^A methylases and demethylases.^[^ ^41^, ^42^ ^]^ First, by probing mTOR level and mTORC1 activity under stiff or soft culture conditions, we observed similar trend of mTOR and phos‐S6 protein changes in cells silenced of METTL3, METTL14, WTAP or VIRMA as in control cells (Figure S4A‐C, Supporting Information), arguing against that these methylases playing a key part in mechano‐sensitive mTOR mRNA m^6^A changes. Next, we interrogated the role of m^6^A demethylases FTO and ALKBH5 using the same strategy. ALKBH5 knockdown had no effect, while FTO knockout (KO) eliminated the difference of mTOR protein and phos‐S6 level under different stiffness conditions (**Figure** **4**A and Figure S4B–E, Supporting Information). The same result was seen in cells treated with the FTO inhibitor—meclofenamic acid (MA; Figure S4F, Supporting Information). To verify the role of FTO in the mechano‐regulation of mTORC1, we monitored mTOR RNA degradation rate in FTO KO cells and observed similar trend between different rigidity states in contrast to WT cells (Figures 4B and 3F), supporting that FTO regulates differential degradation of mTOR transcripts in response to mechanical cues. In addition, we assayed the association of mTOR mRNA with FTO by RIP‐qPCR and found that mTOR mRNA was less enriched by FTO in blebbistatin‐treated cells compared to vehicle control (Figure 4C), suggesting that the interaction between mTOR mRNA and FTO may be disrupted under low contractility, leaving more m^6^A modifications intact without efficient erasing.

We then asked how substrate rigidity impacts FTO. Previous studies reported that FTO had the ability to shuttle between the nucleus and cytoplasm and could be degraded by proteasomes in response to stress.^[^ ^49^, ^50^ ^]^ However, no obvious changes in either the subcellular localization or the protein level of FTO were observed under different rigidities (Figure 4D,E). We then turned to evaluate the recently reported Ser 256 phosphorylation on FTO, which inactivated the protein and blunted its demethylase function.^[^ ^51^ ^]^ The pan‐phosphorylated serine level of FTO was elevated in blebbistatin‐treated cells (Figure 4F). Moreover, when we introduced wild‐type FTO (WT FTO) or an S256D mutant (which mimics the phosphorylated form of FTO) back into the FTO KO cells, we found that only WT FTO but not S256D mutant could restore the global de‐methylated activity via detecting the cellular m^6^A‐transcripts level, as well as the mTOR and phos‐S6 levels in soft cultured cells (Figure 4G,H). Collectively, these results point out a role of FTO post‐translational modification in mechano‐regulation of the mTORC1 pathway.

mTOR appeared to be downregulated by m^6^A methylation (Figure 3), we thus hypothesized that the modified mTOR transcripts were decayed. The m^6^A reader protein YTHDF2 has been reported to mediate the degradation of m^6^A methylated transcripts.^[^ ^52^, ^53^ ^]^ Consistently, knockdown of YTHDF2 prolonged the lifetime of mTOR mRNA, rescued mTOR protein abundance and mTORC1 activity on soft substrate (Figure S4G–I, Supporting Information). These results suggest that YTHDF2 may mediate the downregulation of mTOR mRNA level by decreased matrix rigidity.

Cumulatively, our data uncover that mRNA m^6^A methylation can coordinate with matrix stiffness to regulate mTORC1 pathway via FTO phosphorylation.

### 2.5 GSK3β can Localize to the FA Region and Phosphorylates FTO to Activate mTORC1

Having observed that substrate rigidity influenced FTO phosphorylation, we then set to probe for potential regulators for this post‐translational modification. From immunoprecipitation‐mass spectrometry (IP‐MS), an interaction between Glycogen Synthase Kinase‐3 beta (GSK3β) and FTO was suggested (Figure S4J, Supporting Information). Previous studies have shown that a repeated consensus motif within human FTO “256S/T‐E‐D‐D‐S/T(P)260” matched the GSK3β substrate recognition motif “S/T‐X‐X‐X‐S/T(P)”^[^ ^54^, ^55^ ^]^ (Figure 4I). Thus, we asked whether GSK3β may be the kinase mediating FTO phosphorylation under stiff condition. Indeed, inhibition of GSK3β using CHIR‐98014 decreased the phosphorylation of FTO, while its activation by lowering cellular tension led to the opposite effect (Figure 4F). Meanwhile, agreeing with GSK3β being upstream of the matrix rigidity‐FTO‐mTOR axis, we found that the phos‐S6 level was rescued in cells on soft matrix with CHIR‐98014 treatment (Figure 4J).

We then confirmed the binding between FTO and GSK3β by co‐immunoprecipitation (Co‐IP) in stiff cultured cells (Figure 4K). Next, we interrogated whether and how GSK3β responded to matrix stiffness change. To trace the change of GSK3β localization under different mechanical conditions, we generated a cell line stably expressing GSK3β‐AcGFP. We observed that GSK3β was mostly diffusive in the cytoplasm but displayed plaque‐like structures on the ventral side of the cell, co‐localizing with paxillin at FA sites (Figure S4K, Supporting Information, and Figure 4L). When we forced integrin activation by applying manganese,^[^ ^56^, ^57^ ^]^ we found that the clustered GSK3β distribution at FAs gradually diminished (Figure 4M), suggesting that the FA‐retained GSK3β population may respond to integrin activation and mechanical stimulation. Moreover, manganese increased phosphorylation of GSK3β on Ser9—which indicates its kinase activity inhibition^[^ ^55^, ^58^ ^]^ (Figure 4N). Integrin αVβ3/αVβ5 inhibition upon Cilengitide treatment had no effect on GSK3β activity while silencing integrin β1, which hampered mTORC1 activity at FAs, led to declined phos‐GSK3β (Ser9) level (Figure 4N and Figure S4L, Supporting Information), indicating that GSK3β activity may be negatively regulated by integrin β1. Consistently, we also detected elevated phos‐GSK3β (Ser9) in cells cultured on stiff versus soft substrate but no significant changes in total GSK3β levels (Figure 4O and Figure S4M, Supporting Information), supporting that GSK3β activity was suppressed by substrate stiffening.

Collectively, these results identify the potential role of GSK3β in matrix stiffening‐induced FTO and mTORC1 activation, and that the underlying mechanism may involve integrin β1.

### 2.6 Mechano‐Regulation of mTORC1 Modulates Autophagy During ECM Detachment

**Figure 5:**
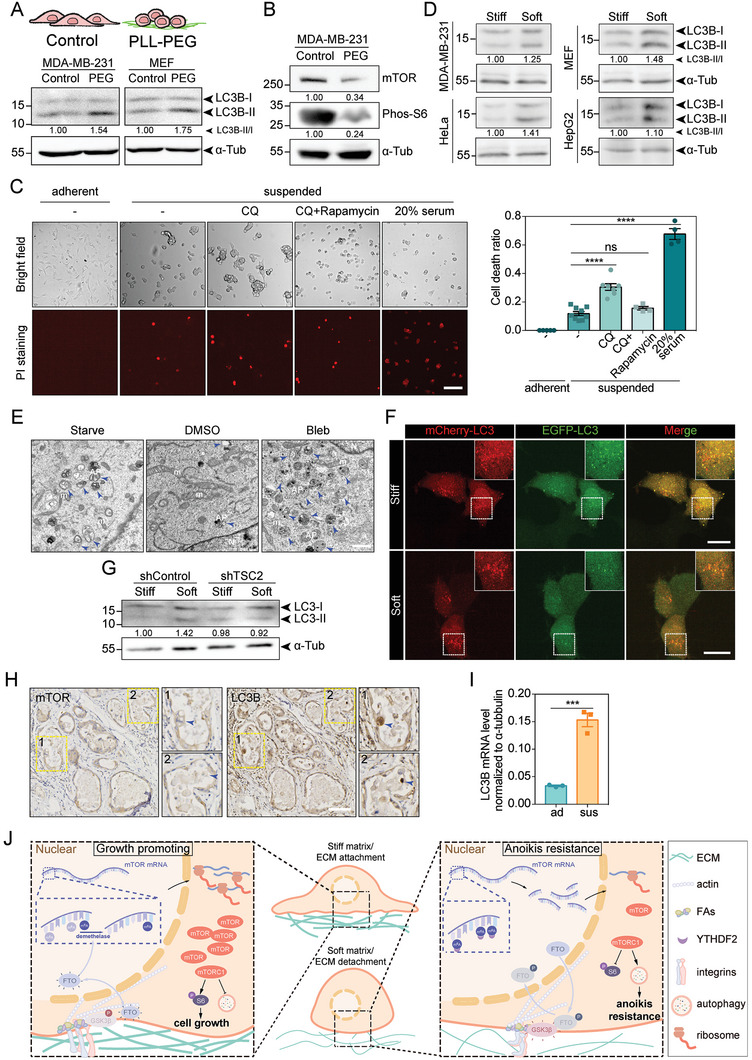
Substrate rigidity modulates autophagy via mTORC1 pathway. A) Top: schematic diagram of cells cultured on pre‐treated substrates. Bottom: western blot showing the LC3‐I and LC3‐II levels in MDA‐MB‐231 and MEF cells cultured on substrates pre‐treated with PBS (Control) or PLL‐PEG. α‐tubulin is used as loading control. B) Western blot showing the mTOR and Phos‐S6 levels in MDA‐MB‐231 cells cultured on PBS (Control) or PLL‐PEG coated substrates. α‐tubulin is used as loading control. C) Left: representative bright field and PI staining images of MDA‐MB‐231 cells under adherent conditions or suspended conditions treated with CQ, CQ+Rapamycin and 20% serum. Right: bar chart shows the quantification of cell death ratio stained by PI (*N*_adherent_ = 5, N_suspended_ = 9, *N*_sus+CQ_ = 7, N_sus+CQ+Rapa_ = 4, *N*_sus+20% serum_ = 4, error bar: mean with SEM, ns = 0.1371, ****p < 0.0001 by unpaired Student's *t*‐test). Scale bar, 150 µm. D) Western blot showing the autophagy maker LC3B levels in MDA‐MB‐231, HeLa, MEF and HepG2 cells cultured on stiff and soft substrates. α‐tubulin is used as loading control. E) Representative TEM images of MDA‐MB‐231 cells treated with DMSO or Bleb. Cells cultured in starved medium were used as positive control. The blue arrow heads indicate autophagosomes. The letter “N” stands for the nucleus and “m” stands for mitochondria. Scale bar, 1 µm. F) Representative images of MDA‐MB‐231 cells expressing tandem mCherry‐EGFP‐LC3B—cultured on stiff and soft substrates. Dashed boxes are zoomed in on the upper right. Scale bar, 20 µm. G) Western blot showing the levels of LC3B‐I and LC3B‐II in shControl and shTSC2 cells cultured on stiff and soft substrates. α‐tubulin is used as loading control. H) Immunohistochemistry images showing the detection of mTOR and LC3B in human breast DCIS sections. The yellow dashed boxes are zoomed in on the right. The blue arrow heads indicate ECM‐detached cancer cells. Scale bar, 200 µm. I) The LC3B mRNA levels of adherent (ad) and suspended (sus) tumor cells in the nude mouse model of malignant ascites (Error bar: mean with SEM, ***p = 0.0006 by unpaired Student's *t*‐test). J) A proposed model for integrin‐GSK3β‐FTO axis mediated mTORC1 regulation in response to different substrate rigidity. Force‐induced integrin β1 activation inhibits GSK3β at the FAs and reduces the phosphorylation on FTO, which is able to erase the m^6^A modification on mTOR transcripts, leading to mTORC1 activation and autophagy inhibition.

ECM detachment, which is equivalent to an extremely soft matrix,^[^ ^5^ ^]^ has been reported to induce metabolic stress that results in anoikis. As a pro‐survival mechanism, autophagy can protect cells from anoikis, facilitating the reattachment to the ECM^[^ ^59^ ^]^ and producing cancer‐promoting effects. When we evaluated LC3B‐II level in MDA‐MB‐231 and MEF cells cultured on low adhesive substrate coated with poly (L‐Lysine)‐PEGs (PLL‐PEG), we observed elevated LC3B‐II (**Figure** **5**A), indicating enhanced autophagy in these detached cells. Meanwhile, we found declined mTOR and phos‐S6 levels in low adhesive substrate (Figure 5B). Next, we observed increased ratio of propidium Iodide (PI) staining, indicative of dead cells, in suspended MEF cells when compared to adherent cells, while chloroquine (CQ), an established pharmacological inhibitor of autophagy, further enhanced the extent of cell death (Figure 5C). Autophagy is under the tight control of mTORC1.^[^ ^60^, ^61^, ^62^ ^]^ Consistent with the inhibitory effect of mTORC1 on autophagy, the enhancement of cell death was significantly blunted in rapamycin‐treated cells. A similar effect of cell death upon CQ treatment was also observed after activating mTORC1 by serum stimulation (Figure 5C). These results suggest that matrix‐detachment is able to enhance autophagy through inhibiting mTORC1 activity, thereby promoting cell resistance to anoikis and improving survival.

LC3B‐II was decreased in cells on stiff substrate or under high contractility while increased in cells on soft substrate or under low contractility, as observed in MDA‐MB‐231, HeLa, MEF, and HepG2 cell lines (Figure 5D and Figure S5A, Supporting Information). Additionally, using transmission electron microscopy (TEM), we observed increased number of autophagosomes in cells under low contractility (Figure 5E and Figure S5B, Supporting Information), indicating a mechano‐regulated autophagy process. Moreover, the autophagic flux was measured by a tandem fluorescent mCherry‐GFP‐LC3 probe,^[^ ^63^ ^]^ and the ratio of GFP/mCherry double positive vesicles decreased in cells under soft culture condition (Figure 5F and Figure S5C, Supporting Information), suggestive of enhanced autophagic flux on soft substrate. When utilizing rapamycin to inhibit mTORC1 activity on stiff substrate, we found an increase in autophagosome number (Figure S5D, Supporting Information). Reciprocally, under blebbistatin treatment, activating mTORC1 pathway by silencing its upstream suppressor‐TSC2 resulted in a reduction of autophagosome formation (Figure S5D,E, Supporting Information). In line with the TEM result, the difference of LC3B‐II level on stiff versus soft substrate was diminished in TSC2 knockdown cells when compared to control cells (Figure 5G), supporting that substrate stiffening hindered autophagy in an mTORC1‐dependent manner.

Given that anoikis resistance is seen during luminal growth in human breast ductal carcinoma in situ (DCIS),^[^ ^64^, ^65^ ^]^ we wondered whether mTOR and autophagy level were changed in these anoikis‐resistant cells. Consistent with this, LC3B level was higher in both detached (inset 1) and luminal cells (inset 2) compared to normal breast epithelium in serially sectioned tissue samples from DCIS patients, while the corresponding mTOR level anti‐correlated with LC3B in detached and luminal cells (Figure 5H). Additionally, we also examined the expression of LC3B and mTOR in mammary gland sections from lactating female mice (Figure S5F, Supporting Information) and, similar scenario was observed in these samples, supporting that ECM detachment evoked autophagy through inhibiting mTORC1 activation.

Epithelial ovarian cancer (EOC) has the highest mortality rate among gynecologic tumors and peritoneal dissemination is the major cause of death in patients with EOC.^[^ ^66^ ^]^ During metastasis, cancer cells detach from the basement membrane and enter the abdominal cavity in danger of anoikis.^[^ ^67^ ^]^ We injected EOC cell line‐A2780 into the cavities of immune‐deficient nude mice to establish malignant ascites model and also injected cells into the flanks as a control. By staining mTOR and LC3B in adherent (isolated from flanks) and suspended (isolated from cavities) A2780 cells, we found a significant decrease of mTOR immunofluorescent intensity in suspended cells compared to adherent cells (Figure S5G, Supporting Information), while LC3B intensity and mRNA level appeared an opposite trend (Figure 5I and Figure S5H, Supporting Information). These results further suggest that matrix detachment‐regulated mTORC1‐autophagy pathway is involved in the progression of EOC.

## 3 Discussion

Mechanical force widely participates in various biological processes including cell proliferation, differentiation and migration that require metabolic rewiring.^[^ ^4^, ^68^, ^69^ ^]^ As the major regulator of anabolism, mTORC1 has been the highlight of research in cell growth and tumor survival, however, an association of it with the mechano‐microenvironment is largely missing. Here, we demonstrate an integrin‐GSK3β‐FTO‐mTOR axis which is downregulated under soft conditions and influences autophagy (Figure 5J).

Integrins‐based FAs are dynamic protein complexes that mediate the association of the actin cytoskeleton with the ECM^[^ ^24^, ^70^ ^]^ and are functionally involved in cell polarization, spreading, and migration via canonical mechano‐transduction pathway. Recent emerging evidence has demonstrated the distribution of growth factor receptors and amino acid transporters at FA sites.^[^ ^39^ ^]^ In addition, localized protein translation has been found in the FA‐enriched cell area.^[^ ^71^ ^]^ New roles of FAs above mechano‐sensing are to be unveiled. Here, we showed mTOR and phos‐S6 can promote cell proliferation and inhibit autophagy through local activation at the integrin‐FA sites. This activation depends on the substrate sensing, cellular contraction as well as integrin β1. These observations concertedly suggest a role of integrin‐conjugated FAs in modulating cellular metabolism.

We identified FTO‐one of the m^6^A demethylases being crucial for the mechano‐regulation of mTOR mRNA and subsequent protein abundance. Recently, post‐translational modifications (PTMs) of FTO have attracted increasing attention. It has been reported that the 150 phosphorylation significantly changed FTO's subcellular localization and substrate specificity.^[^ ^72^, ^73^ ^]^ Additionally, phosphorylation by protein kinase Cβ (PKCβ) directed FTO towards ubiquitin‐mediated protein degradation.^[^ ^49^ ^]^ Here, we found that phosphorylation at Ser 256 hampered FTO catalytic activity without affecting its subcellular localization or protein abundance, suggesting that PTMs influence FTO through various ways. One possibility of altered FTO activity under different stiffness is that phosphorylation modifies FTO activity by previously reported enhancement of Ca^2+^ binding.^[^ ^74^ ^]^ Another possibility is that phosphorylation status changes the FTO activity through influencing its oligomerization, however, determination of the exact impact of the dimerization on the FTO catalytic activity is not easy, due to the structure complexity and technical limitations.^[^ ^75^ ^]^

The impact of ECM stiffening on tumor growth and aggressiveness is widely acknowledged, with various mechanisms being implicated including suppression of immune response in tumors,^[^ ^76^ ^]^ providing tracks for cancer cell invasion, or promoting exosome secretion,^[^ ^2^, ^77^ ^]^ etc. When attached to ECM, tumor cells sense the substrate rigidity via the integrin‐FA complex. However, they frequently encounter environmental changes during metastasis, such as detachment from the ECM due to loss of adhesion in cavity or ductal tissues, which can induce anoikis‐mediated cell death. Acquisition of molecular strategies that confer anoikis resistance is a critical step in oncogenesis. Interestingly, our study has revealed that the mechano‐activation of the mTORC1 pathway may play a dual‐role in both stiffness promoted cell growth and resistance to anoikis. Increased substrate stiffness or cell tension enhance mTORC1 activation, maintaining cells proliferation with anabolic metabolism. While ECM detachment inactivates mTORC1 signaling and actually promotes cell survival by elevating autophagy. How tumor cells harness this machinery to navigate through changes of the mechano‐microenvironment and how this pathway could contribute to targeted therapy awaits future investigations.

## 4 Experimental Section

### Plasmids, siRNA, and shRNA

All plasmids were constructed using BM seamless cloning kit to ligate PCR products into a backbone vector opened by restriction digestion. Human FTO and GSK3β were cloned from the MDA‐MB‐231 cell‐extracted cDNA library. Plasmid containing LC3B was purchased from Addgene (#73949). FTO WT, S256D, GSK3β‐GFP were subcloned into lentiviral vector (pLVX‐AcGFP‐N1) using EcoRI and BamHI enzymes from Transgene (JE201‐01, JB101‐01) for establishing AcGFP‐fused plasmids. For S256D, FTO gene was mutated at Ser 256 site using Site‐directed Gene Mutagenesis Kit (beyotime, D0206). For mCherry‐EGFP‐LC3B, mCherry and EGFP fragments were amplified by regular PCR and then inserted into N‐terminus of LC3B gene on pLVX‐AcGFP‐N1 vector using EcoRI and XbaI enzymes (Transgene, JX101‐01). For Flag‐FTO, FTO was fused with a Flag tag contained in a short PCR primer into pLVX‐AcGFP‐N1 backbone.

shRNA

shControl: 5′‐AACGCTGCTTCTTCTTATTTA‐3′;

shITGB1‐1: 5′‐GCTCAAGCCAGAGGATATTAC‐3′;

shITGB1‐2: 5′‐GGATATTACTCAGATCCAACC‐3′;

shTSC2‐1: 5′‐GCATGGAATGTGGCCTCAACA‐3′;

shMettl3: 5′‐GCCAAGGAACAATCCATTGTT‐3′;

shMettl14: 5′‐GGATGAACTAGAAATGCAACA‐3′;

shWTAP: 5′‐GTTATGGCAAGAGATGAGTTA‐3′;

shVIRMA: 5′‐GGAGTTATATCAAGAGGAAAG‐3′;

shALKBH5: 5′‐GCTTCAGCTCTGAGAACTACT‐3′;

shYTHDF2: 5′‐GATGGATTAAACGATGATGAT‐3′;

shFTO‐1: 5′‐TCACCAAGGAGACTGCTATTT‐3′;

shFTO‐2: 5′‐CGGTTCACAACCTCGGTTTAG‐3′;

shmTOR‐1: 5′‐TTTGAGCATGCCGTCAATAA‐3′;

shmTOR‐2: 5′‐GCTGTGCTACACTACAAACAT‐3′.

siRNA

scramble: sense 5′‐UUCUCCGAACGUGUCACGUTT‐3′;

scramble: antisense 5′‐ACGUGACACGUUCGGAGAATT‐3′;

vinculin‐1: 5′‐GGAAGAAAUCACAGAAUCAUU‐3′;

vinculin‐2: 5′‐CCAGAUGAGUAAAGGAGUAUU‐3′;

paxillin‐1: 5′‐CCCUGACGAAAGAGAAGCCUA‐3′

paxillin‐2: 5′‐UAGGCUUCUCUUUCGUCAGGG‐3′

ITGAV‐1: 5′‐CGACAAAGCTGAATGGATT‐3′;

ITGAV‐2: 5′‐GCTTAAAGGCAGATGGCAA‐3′;

ITGA1‐1: 5′‐TGGCAAGACTATAAGGAAA‐3′;

ITGA1‐2: 5′‐TCACAGAAGTAAAGGAGAA‐3′;

ITGA3‐1: 5′‐GGACAACCTCCGTGACAAA‐3′;

ITGA3‐2: 5′‐GCTACATGATTCAGCGCAA‐3′;

ITGA5‐1: 5′‐CCTCAATGCTTCTGGAAAA‐3′;

ITGA5‐2: 5′‐GGATAGAGGACAAGGCTCA‐3′;

ITGA8‐1: 5′‐GAAACTGAATTCCGAGATA‐3′;

ITGA8‐2: 5′‐GAACCAAGATGGATACAAT‐3′;

ITGB1‐1: 5′‐GCGAGUGUGAUAAUUUCAA‐3′;

ITGB1‐2: 5′‐UUGAAAUUAUCACACUCGC‐3′;

ITGB3‐1: 5′‐CCUGCACCUUUAAGAAAGA‐3′;

ITGB3‐2: 5′‐GTTGATGCTTATGGGAAAA‐3′;

ITGB5‐1: 5′‐AGAAATTGGCAGAGAACAA‐3′;

ITGB5‐2: 5′‐GCACCAAACTCGCGGAGGA‐3′;

ITGB6‐1: 5′‐AGAAAGAAGTGGAAGTGAA‐3′;

ITGB6‐2: 5′‐CCAAAGAGATGTCTAAATT‐3′;

ITGB8‐1: 5′‐GCAGAAACGTGACGAGCAA‐3′;

ITGB8‐2: 5′‐TGAGAAGCCTGAAGAAATA‐3′.

sgRNA

FTO‐1: 5′‐GAAGCGCACCCCGACTGCCG‐3′;

FTO‐2: 5′‐CTCTCGTTCCTCGGCAGTCG‐3′;

FTO‐3: 5′‐AGCTTCGCGCTCTCGTTCCT‐3′.

qPCR primer sequences

mTOR‐F: 5′‐AGCATCGGATGCTTAGGAGTGG‐3′;

mTOR‐R: 5′‐CAGCCAGTCATCTTTGGAGACC‐3′;

LC3B‐F: 5′‐GAGAAGCAGCTTCCTGTTCTGG‐3′;

LC3B‐R: 5′‐GTGTCCGTTCACCAACAGGAAG‐3′;

Mettl3‐F: 5′‐CTATCTCCTGGCACTCGCAAGA‐3′;

Mettl3‐R: 5′‐GCTTGAACCGTGCAACCACATC‐3′;

Mettl14‐F: 5′‐CTGAAAGTGCCGACAGCATTGG‐3′;

Mettl14‐R: 5′‐CTCTCCTTCATCCAGATACTTACG‐3′;

WTAP‐F: 5′‐GCAACAACAGCAGGAGTCTGCA‐3′;

WTAP‐R: 5′‐CTGCTGGACTTGCTTGAGGTAC‐3′;

VIRMA‐F: 5′‐TGACCTTGCCTCACCAACTGCA‐3′;

VIRMA‐R: 5′‐AGCAACCTGGTGGTTTGGCTAG‐3′;

TSC2‐F: 5′‐GCACCTCTACAGGAACTTTGCC‐3′;

TSC2‐R: 5′‐GCACCTGATGAACCACATGGCT‐3′.

α‐Tub‐F: 5′‐CGGGCAGTGTTTGTAGACTTGG‐3′

α‐Tub‐R: 5′‐CTCCTTGCCAATGGTGTAGTGC‐3′

### Antibodies and Reagents

The following antibodies were used in this study: mouse anti‐α‐tubulin (T9026, 1:5000 for western blotting) from Sigma‐Aldrich; mouse anti‐paxillin (610620, 1:200 for IF) from BD Biosciences; mouse anti‐AMPKalpha (F6) (2793S, 1:1000 for western blotting and IHC), Rabbit anti‐mTOR (2983S, 1:4000 for western blotting and 1:200 for IF) and Rabbit anti‐P‐S6 Ribosomal (2211S, 1:4000 for western blotting and 1:200 for IF) from Cell Signaling Technology; rabbit anti‐METTL3 (A8370, 1:1000 for western blotting), rabbit anti‐Phos‐S6 Ribosomal (AP0538, 1:1000 for western blotting), rabbit anti‐Phos‐GSK3β (S9) (AP1088, 1:1000 for western blotting), rabbit anti‐FTO (A3861, 1:1000 for western blotting and 1:200 for IF), rabbit anti‐ALKBH5 (A11684, 1:1000 for western blotting), rabbit anti‐integrin β1 (A19072, 1:1000 for western blotting), rabbit anti‐mTOR (A11355, 1:1000 for western blotting), rabbit anti‐pan‐Phos‐Serine (AP0932, 1:1000 for western blotting), rabbit anti‐DEPDC6 (A9447, 1:1000 for western blotting), rabbit anti‐MLST8 (A13599, 1:1000 for western blotting), rabbit anti‐β‐catenin (A19657, 1:1000 for western blotting); rabbit anti‐Raptor (A8992, 1:1000 for western blotting) from ABclonal; rabbit anti‐GAPDH (ab181602, 1:4000 for western blotting) from Abcam; YAP (sc‐101199, 1:200 for IF); Secondary antibodies used for western blot in this study: mouse anti‐GFP (M048‐3, 1:4000 for western blotting and 1 uL antibody per milligram protein for Co‐IP) from MBL International; anti‐mouse (sc‐516102, 1:4000) and anti‐rabbit (sc‐2004, 1:4000) horseradish peroxidase (HRP)‐conjugated secondary antibodies from Santa Cruz Biotechnology. Secondary antibodies used for immunofluorescent staining in this study: Donkey anti‐Rabbit IgG (H+L) Highly Cross‐Adsorbed Secondary Antibody, Alexa Fluor 488 (Life Technologies, A21206, 1:200) and Donkey anti‐Mouse IgG (H+L) Highly Cross‐Adsorbed Secondary Antibody, Alexa Fluor 555 (Life Technologies, A31570, 1:200) and Goat anti‐Mouse IgG (H+L) Highly Cross‐Adsorbed Secondary Antibody, Alexa Fluor 647 (Life Technologies, A21236, 1:200).

The following reagents were used in this study: Dimethyl sulfoxide (DMSO) (Amresco, 0231–500ML); Blebbistatin (EMD Millipore, 2 946 047); MG132 (EMD Millipore, 474790‐20MG); Rapamycin (Selleck, S1039); Cilengitide (Selleck, S7077); CHIR‐98014 (Selleck, S2745); Meclofenamate Sodium (MA) (Selleck, S4295); Phosphatase inhibitor cocktail 3 (Sigma‐Aldrich, P0044); Cycloheximide (CHX) (Sigma‐Aldrich, C7698); Manganese Chloride (Sigma‐Aldrich, M1787); Fibronectin (Sigma‐Aldrich, F1056‐5MG); Propidium iodide (PI) (Sigma‐Aldrich, P4170‐10 mg); Puromycin (Sigma‐Aldrich, P8833); MES (2‐(N‐Morpholino) ethanesulfonic acid, 4‐Morpholineethanesulfonic acid) (Sigma‐Aldrich, M3671‐50G); Protease inhibitor mini tablets (Thermo Fisher Scientific, 88 666); EDC (1‐Ethyl‐3‐(3‐dimethylaminpropyl) carbodiimide HCl) (Thermo Fisher Scientific, 22 980); NHS (N‐Hydroxysuccinimide) (Thermo Fisher Scientific, 24 500); Trizol (Life Technologies, 15 596 026); ActinomycinD (Bioss, D50409s); PLL‐PEG (Nanosoft Polymers); Chloroquine (CQ) (Leyan, 1 062 181); Rho activator II (Cytoskeleton, CN03); Polybrene (Macgene, MC032); Y27632 (Cell Signaling Technology, 136424S); Neofect (Genomtech, TF20121201); RNATransMate (Sangon Biotech, E607402‐0100); Protein A+G beads (Beyotime, P2012); Anti‐Flag (DYKDDDDK) Affinity gel (Bimake, 23 102); Anti‐GFP Affinity beads (Smart‐Lifesciences, SA070001); Cell Counting Kit‐8 (CCK‐8) (Dojindo, CK04).

In addition to the usage mentioned in figure legend, blebbistatin was used at the concentration of 30 µM for 6–8 h. Rapamycin was used at the concentration of 40 nM for 24 h. Rho activator II was used at the concentration of 1 mg/mL for 6–8 h. Cilengitide was used at the concentration of 5 mM for 6 h. Y‐27632 was used at the concentration of 10 mM for 2 h. MG132 was used at the concentration of 20 µM and CHX was used at the concentration of 10 µg mL^−1^. Actinomycin D was used at the concentration of 20 µM and CHIR‐98014 was used at the concentration of 2 µM for 6 h. Manganese Chloride was used at the concentration of 1 µM for 6 h. PLL‐PEG was used at the concentration of 5 µg mL^−1^. CQ was used at the concentration of 25 µM for 24 h. MA was used at the concentration of 100 µM for 6–8 h. PI was used at the concentration of 5 µM for 0.5 h.

### Cell Lines

Mouse embryonic fibroblasts (MEFs) were generously provided by James Bear laboratory (University of North Carolina at Chapel Hill, USA). NIH3T3 cells were generously provided by Mo Li laboratory (Third Hospital, Peking University, China); Human embryonic kidney 293T (HEK 293T) cells and HeLa cells were generous gifts from Yuxin Yin laboratory (Peking University, China). MDA‐MB‐231 cells and human retinal pigment epithelium (HRPE) cells were generously provided by Yujie Sun laboratory (Peking University, China). A2780 cells were generous gifts from Congrong Liu laboratory (Third Hospital, Peking University, China). HepG2 cells were generously provided by Fengmin Lu laboratory (Peking University, China). Cells were cultured in Dulbecco's modified Eagle medium (DMEM; Corning, 10‐013‐CRVC) supplemented with 10% fetal bovine serum (FBS; PAN‐Biotech, P30‐3302), 100 U mL^−1^ penicillin and 100 mg/mL streptomycin at 37 °C with 5% CO_2_. In conditions of starvation, cells were maintained in DMEM supplemented with 1% FBS. For cell passage, cells were washed with DPBS (Macgene, CC010) and digested with trypsin (Macgene, CC012).

### CRISPR/Cas9‐Mediated FTO Gene Knockout

FTO knockout cells were constructed by CRISPR‐Cas9 system. Specific guide RNA was ligated into the BsmB1 restriction site of the lentiviral vector (lentiCRISPR v2‐Puro). Lentivirus particles were produced by co‐transfected HEK293T with guide RNA plasmids, packaging plasmids pCMV‐VSV‐G and psPAX2. The medium was changed to fresh DMEM containing 20% FBS at 24 h post transfection and viral supernatant was collected at 48–72 h. MDA‐MB‐231 cells were infected 3 times with 1 mL viral supernatant, 1 mL DMEM supplemented with 2 µL polybrene and incubated at 37°C for 12–24 h. Possible knockout MDA‐MB‐231 cells were screened by puromycin at 2 µg/mL for 1 week and each monoclone was confirmed by DNA sequencing.

### Cell Viability Assay

2000 cells were seeded per well in 96‐well plates and spread for 12 h then performing drugs treatments. Cells were further incubated for 24 h, 48 h, or 72 h, exchanged for fresh medium every 24 h and treated with the same drugs. Samples with different conditions plot growth curves by counting cells at different time points. Cells were incubated with CCK8 reagent at 37°C for 1 h, and then assayed by 450 nm absorbance measurement. The cell viability under each condition was then quantified from the absorbance and normalized to control.

### Western Blot

For western blotting, cells were washed with DPBS once and lysed in an appropriate volume of RIPA buffer (50 mM Tris‐HCl, pH 8.0, 150 mM NaCl, 1% Triton X‐100, 0.5% Na‐deoxycholate, 0.1% SDS, 1 mM EDTA and protease inhibitor cocktail) for 20 min on ice. Lysates were centrifuged at 13,572 g for 10 min and the supernatants were collected. Then, 5× SDS loading buffer was added to the supernatants and boiled for 10 min at 95°C. Protein samples were run on 10% SDS–PAGE acrylamide gels and transferred onto nitrocellulose (NC) membranes by wet electrophoretic transfer, followed by primary and second antibody incubation at 4°C overnight or room temperature for 2 h. The X‐ray film were used to detect and record the band intensities. The fixed X‐ray film was scanned and digital images were obtained. The images were processed by ImageJ software (https://imagej.nih.gov/ij/). Images were first transferred to 8‐bit depth. Then, the intensity of bands was quantified using ImageJ (ImageJ→Analyze→Gels).

### Immunofluorescence

Cells were plated on acid‐washed coverslips coated with or without 10 µg/mL fibronectin at 37 °C for 1 h. Cells were then fixed with 4% paraformaldehyde at room temperature for 15 min, permeabilized in 0.5% Triton X‐100 in DPBS for 8 min, and blocked with 5% bovine serum albumin (BSA) for 1 h. To stain target proteins, the primary antibodies were diluted 1:200 in DPBS and incubated for 1 h at room temperature. After washing with DPBS three times, the coverslips were incubated with Alexa Fluor 488, 555, or 647‐conjugated secondary antibody for 1 h at room temperature. After another wash with PBS three times, the coverslips were mounted with ProLong Glass Antifade Mountant with NucBlue Stain (P36981, Invitrogen). After mounting medium was solidified, images were captured by Andor Dragonfly confocal imaging system and FACILITY STED microscopy (Abberior).

### Co‐Immunoprecipitation

Cells were lysed with IP lysis buffer (25 mM Tris (pH 7.4), 1 mM EDTA, 150 mM NaCl, 5% glycerol, 1% NP‐40, pH 7.4) for 15 min on ice. The cell lysis was centrifuged at 13572 g for 10 min at 4 °C to remove the insoluble components. 80 µL of lysates were taken into a clean microfuge tube as “input” samples. The other supernatants were added with antibodies (1 µg antibody per milligram protein) and rotated at 4 °C overnight. Then the protein A and G beads were washed with IP lysis buffer and added to the antibodies‐supernatants mixtures followed by 3 h rotating at 4 °C. Finally, the beads were collected and washed three times with lysis buffer and boiled in 1x SDS sample buffer. 5x SDS loading buffer was used to boil the “input” samples. To detect the interaction proteins, the samples were separated by SDS‐PAGE gels. To analyze protein interactome, the samples were separated by SDS‐PAGE gels then the target gels were collected for mass spectrum.

### Fabrication of Uniform Polyacrylamide (PA) Gels

Uniform PA gels were made from 40% acrylamide and 2% bis‐acrylamide mixed with 10% ammonium persulfate and 1% TEMED, where varying ratios of acrylamide and bis‐acrylamide were used to create gels of known reproducible stiffness.^[^ ^78^ ^]^ 80 µL acrylamide mix was applied to glutaraldehyde‐modified 24 mm glass coverslip, covered with a glass coverslip made hydrophobic by treatment with Repel‐Silane. After gel gelation, the hydrophobic glass coverslip was removed and the gel was washed with PBS thoroughly to remove unreacted reagents. The stiffness was measured with atomic force microscopy (AFM). To promote cell adhesion, fibronectin was covalently linked to the gels as described below.

### Functionalizing PA Gel with Fibronectin

Premixed solutions (Soak solution: 137 mM NaCl, 5% glycerol; 2x conjugation buffer: 0.2 M MES, 10% glycerol, pH 4.5; 10x EDC: 150 mM, 29 mg mL^−1^ in DI water; 10x NHS: 250 mM, 29 mg mL^−1^ in DI water) were prepared. Soak solution was first pipetted to each dish such that the gel was completely submerged, then dishes were incubated at room temperature for at least 30 min. After that, 1 part 10x EDC, 1 part 10x NHS, 3 parts DI water, and 5 parts 2x conjugation buffer were mixed together. All soak solution from dishes was removed, then enough NHS/EDC solution was added to cover the gel surface and dishes were incubated at room temperature or 37°C for 30 min in the darkness. Then NHS/EDC solution was discarded and 10 µg mL^−1^ fibronectin solution diluted in PBS was added to the gels, then the gels were incubated at 37 °C for 1 h.

### Dual‐Luciferase Reporter Assay

HEK293T and MDA‐MB‐231 cells seeded in 24‐well plate were transiently transfected with 0.1 µg *Renilla* reporter plasmid together with 2 µg luciferase plasmid containing mTOR promoter for 48 h. Cells were lysed with Dual‐luciferase Reporter Detection Kit (Yuanpinghao Bio., GN201‐01), then reporter gene activity was analyzed using the Dual‐Luciferase Reporter 1000 Assay System (Promega, E1960) and measured with a TD‐20/20 Luminometer (Turner Designs) according to the manufacturers' instructions.

### Transmission Electron Microscopy (TEM)

Cells were seeded on the ACLAR Films (50 425) in 24‐well plates and cultured for 12 h before fixation. Then cells were washed with 37 °C PB buffer (0.2 M NaH_2_PO_4_ and Na_2_HPO_4_, pH  =  7.2–7.4), and immediately fixed with PB buffer containing 2% PFA and 2.5% glutaraldehyde at room temperature for 1 h and 4 °C overnight. After post‐fixation in 1% osmium tetroxide and pre‐embedding staining with 1% uranyl acetate, samples were dehydrated and embedded in SPI‐Pon 812 resin. TEM images were acquired using Jeol JEM‐1400 electron microscope operated at 80 kV.

### Mass Spectrometry

For protein identification, the Coomassie‐stained total aggregated proteins of each sample were cut out of the gel and destained with a solution of 100 mM ammonium bicarbonate in 50% acetonitrile. After dithiothreitol reduction and iodoacetamide alkylation, the proteins were digested with porcine trypsin (Sequencing grade modified; Promega, Madison, WI) overnight at 37°C. The resulting tryptic peptides were extracted from the gel pieces with 80% acetonitrile, 0.1% formic acid (FA). The samples were dried in a vacuum centrifuge concentrator at 30 °C and resuspended in 10 µL 0.1% FA. Using an Easy‐nLC 1200 system, 5 µL of sample were loaded at a speed of 0.3 µL mi^−1^n in 0.1% FA onto a trap column (C18, Acclaim PepMap TM 100 75 µm × 2 cm nanoViper Thermo) and eluted across a fritless analytical resolving column (C18, Acclaim PepMap TM 75 µm × 25 cm nanoViper RSLC Thermo) with a 75‐min gradient of 4 to 90% LC‐MS buffer B (LC‐MS buffer A includes 0.1% formic acid; LC‐MS buffer B includes 0.1% formic acid and 80% ACN) at 300 nL mi^−1^n. Peptides were directly injected into a Thermo Orbitrap Exploris 480 with FAMIS pro using a nano‐electrospray ion source with electrospray voltages of 2.2 kV. Full scan MS spectra were acquired in the Orbitrap mass analyzer (m/z range: 350–1500 Da) with the resolution set to 60000 (FWHM) at m/z 200 Da. Full scan target was 300% with a maximum IT of 30 ms. All data were acquired in profile mode using positive polarity. MS/MS spectra data were acquired in the Orbitrap as well with a resolution of 15000 (FWHM) at m/z 200 Da and AGC target value for fragment spectra was set at 100% with a maximum IT of 22 ms. Isolation width was 1.6 m/z. Normalized collision energy was set at 30%. FAIMS CV were set as ‐45 V and ‐65 V. The MS data were aligned with UniProtKB/Swiss‐Prot reviewed human proteome database.

### Anoikis Assay

Glass slides were coated with PLL‐PEG (5 µg mL^−1^ in PBS) for 1 h at 37 °C to prevent cells from adhering to the base of culture dishes. This was performed to mimic the anchorage‐independent growth conditions of the cells.

### Mice and Tumor Xenograft Model

Animal studies were conducted according to guidelines approved by the Biomedical Ethics Committee of Peking University (approval no. LA2020398). Female BALB/c nude (6‐week‐old) mice were maintained in specific pathogen‐free conditions at the Center of Experimental Animals (Peking University, Beijing, China). A2780 cells (3 × 10^6^) were injected into the left flank and right flank, respectively. Then, 5 × 10^6^ A2780 cells were injected into the peritoneal cavity to establish malignant ascites mice models. Animals were then monitored for tumors growth. After four weeks, animals were sacrificed. Tumors from the left and right flanks were collected and digested to extract RNA for qPCR. DPBS was used to lavage the peritoneal cavities of mice and ascite cells were centrifuged and collected for smear and immunofluorescence staining, or RNA extraction for qPCR. All protocols in this study were performed in accordance with the Declaration of Helsinki.

### Patient Samples and Immunohistochemistry (IHC)

Human breast DCIS samples were purchased from Xi'an bioaitech Co., Ltd (Xi'an, China). The samples were analyzed by IHC assay using anti‐mTOR or LC3B antibody according to standard method.

### Mammary Lactate Gland Samples

Lactating mammary glands were obtained from BALB/c female mice. The samples were analyzed by IHC assay using anti‐mTOR or LC3B antibody according to standard method.

### RNA Sequencing

The total RNA was extracted using Trizol according to the manufacturer's instructions. RNA was qualified and quantified as follows: (1) RNA purity and concentration were then examined using NanoDrop 2000; (2) RNA integrity and quantity were measured using the Agilent 2100/4200 system. RNA library for RNA‐seq was prepared as flows: mRNA was purified from total RNA using polyT and then fragmented into 300–350 bp fragments, the first strand cDNA was reverse‐transcribed using fragmented RNA and dNTPs (dATP, dTTP, dCTP and dGTP) and second strand cDNA synthesis was subsequently performed. Remaining overhangs of double‐strand cDNA were converted into blunt ends via exonuclease/polymerase activities. After adenylation of 3′ ends of DNA fragments, sequencing adaptors were ligated to the cDNA and the library fragments were purified. The template was enriched by PCR, and the PCR product was purified to obtain the final library. After library preparation and pooling of different samples, the samples were subjected for Illumina sequencing. Raw data (raw reads) of FASTQ format were first processed through in‐house perl scripts. In this step, clean data (clean reads) were obtained by removing following reads: (1) reads with adaptor; (2) reads with more than 3 N; (3) reads with more than 20% nucleotides with Qphred < = 5; At the same time, Q20, Q30 and GC content of the clean data were calculated. Then, map the clean reads to the silva database to remove the rRNA. All the downstream analyses were based on the clean data without rRNA. Paired‐end clean reads were aligned to the reference genome using Hisat2. Featurecount was used to count the reads numbers mapped to each gene. DESeq2 was used for differential expression analysis. KEGG and MigDB were used for enrichment analysis of differentially expressed gene sets.

### Real‐Time Quantitative PCR (RT‐qPCR)

Total RNA from MDA‐MB‐231 cells were isolated using Trizol. RNA was reverse transcribed using a Transcript One‐Step gDNA Removal and cDNA Synthesis SuperMix Kit (Transgene, AT311‐02). Level of the mTOR, LC3B, FTO and α‐Tubulin genes were analyzed by RT‐qPCR amplified using SYBR Green (ABclonal, RK21203). Data shown are the relative abundance of the target mRNA normalized to α‐tubulin. The primer sequences used for qPCR were listed in the table.

### MeRIP‐qPCR

Total RNA of cells with specific treatment were isolated using Trizol and then enriched for mRNA. Target m^6^A‐containing fragments were pulled down using a beads‐bound m^6^A capture antibody, and RNA sequences in both ends of the m^6^A‐containing regions were cleaved using Cleavage Enzyme Mix. The enriched RNA was then released, purified and eluted. qPCR was performed after MeRIP to quantify changes of predicted m^6^A sites in target gene. MeRIP‐qPCR assay and subsequent data analysis were supported by GeneSky Biotechnologies Inc. (Shanghai, China).

### RIP‐qPCR

After co‐immunoprecipitation with anti‐FTO antibody, the co‐precipitated RNAs were isolated and purified using Trizol from the beads and dissolved in RNase‐free water. Binding RNA targets were analyzed using qPCR.

### In Vitro RNA Methylation Assay

For quantification of m^6^A levels, 200–250 ng total RNA extracted from cells with different treatment were used to measure the cellular m^6^A levels using the EpiQuik m^6^A RNA Methylation Quantification kit (colorimetric) (Epigentek, P‐9005) according to the manufacturer's protocols.

### Polysome Profiling

MDA‐MB‐231 cells in DMSO and blebbistatin groups were treated with 100 µg mL^−1^ cycloheximide (CHX) for 15 min and then digested with trypsin‐EDTA and centrifuged in DPBS. The cell pellets were collected and flash freeze in liquid nitrogen. Polysome profiling assay was conducted by Chi‐biotech (Shen Zhen, China). Total RNA was purified from the several ribosome fractions using TRIzol reagent and then RT‐PCR was conducted to analyze mTOR mRNA level.

### Statistics and Data Display

The number of biological and technical replicates and the number of samples are indicated in figure legends and the main text. Data are mean ± SEM, ± SD or 95% CI. as indicated in the figure legends and supplementary figure legends. Paired/unpaired Student's *t*‐test and one‐way *ANOVA* were performed with GraphPad Prism 7.0 and Excel (Microsoft). Data from image analysis was graphed using Prism 7.0.

## Conflict of Interest

The authors declare no conflict of interest.

## Author Contributions

C.Z. and Y.W. contributed equally to this work. C.W., C.Z., and Y.W. conceived the project and designed the experiments. C.Z., Y.W., and Z.Z. performed the experiments and data analysis. J.L. assisted with the construction of the plasmids and western blot. J.S. performed IHC assay of mice samples. All authors participated in discussion and editing of the manuscript.

## Supporting information

Supporting Information

## Data Availability

The data that support the findings of this study are available from the corresponding author upon reasonable request.
