# Supplementary material for: mTORC1 Mediates Biphasic Mechano‐Response to Orchestrate Adhesion‐Dependent Cell Growth and Anoikis Resistance
Source: Adv Sci (Weinh). 2023 Dec 2;11(6):2307206. doi: 10.1002/advs.202307206 (PMC10853740; doi:10.1002/advs.202307206)
Supplement: Supplementary file 1 — Supporting Information [file ADVS-11-2307206-s001.pdf]

ADVANCED SCIENCE
Open Access

Supporting Information

for *Adv. Sci.*, DOI 10.1002/advs.202307206

mTORC1 Mediates Biphasic Mechano-Response to Orchestrate Adhesion-Dependent Cell Growth and Anoikis Resistance

*Chunlei Zhang, Yuan Wang, Zifeng Zhen, Jiayi Li, Jing Su and Congying Wu**

Supplementary Materials for

• mTORC1 mediates biphasic mechano-response to orchestrate adhesion-dependent cell growth and anoikis resistance

Chunlei Zhang *et al.*

*Corresponding author: Congying Wu, congyingwu@hsc.pku.edu.cn

This PDF file includes:

Figs. S1 to S5
Legends for Figs. S1 to S5

Figure S1

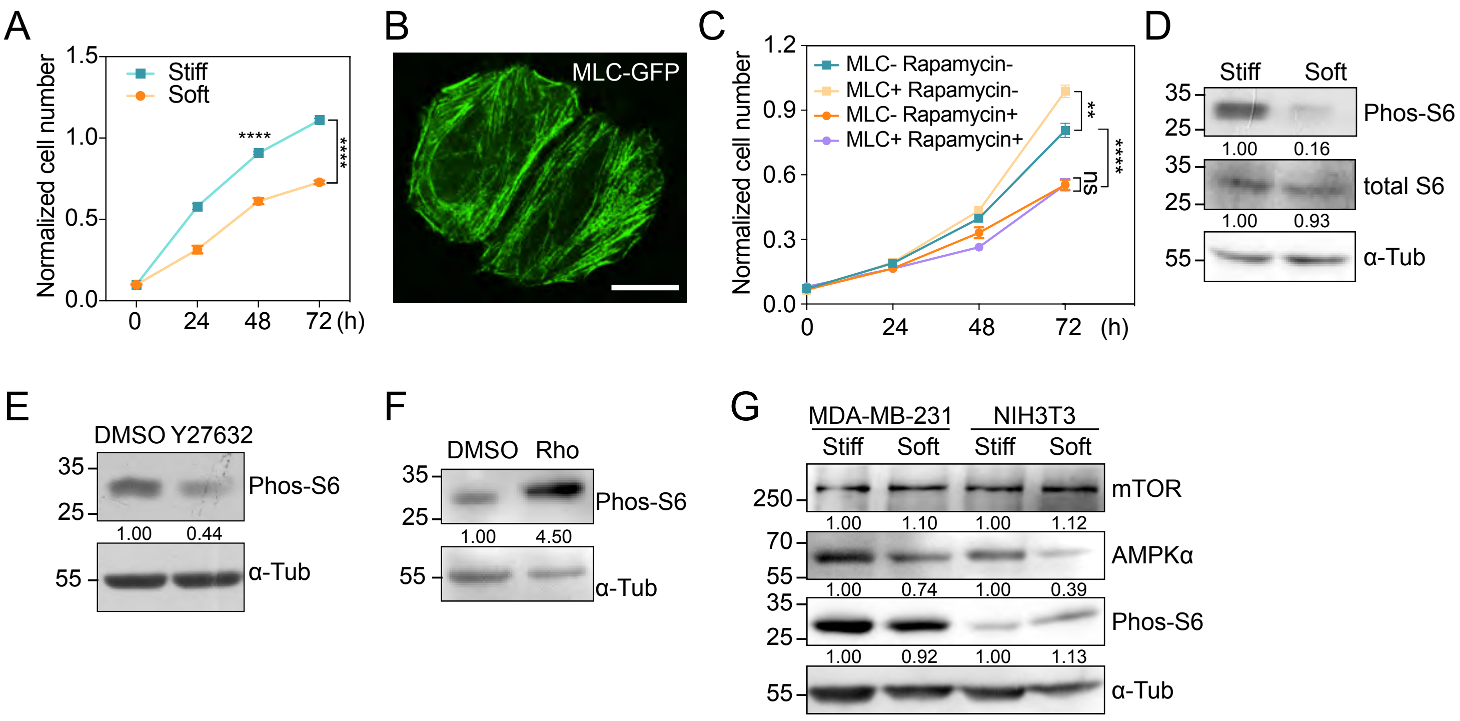

A
Normalized cell number
1.5
1.0
0.5
0.0
Stiff
Soft
****
****
0
24
48
72 (h)
B
MLC-GFP
C
1.2
0.9
0.6
0.3
0.0
Normalized cell number
MLC- Rapamycin-
MLC+ Rapamycin-
MLC- Rapamycin+
MLC+ Rapamycin+
**
****
ns
0
24
48
72
(h)
D
Stiff
Soft
35
25
Phos-S6
1.00
0.16
35
25
total S6
1.00
0.93
55
α-Tub
E
DMSO
Y27632
35
25
Phos-S6
1.00
0.44
55
α-Tub
F
DMSO
Rho
35
25
Phos-S6
1.00
4.50
55
α-Tub
G
MDA-MB-231
NIH3T3
Stiff
Soft
Stiff
Soft
250
mTOR
1.00
1.10
1.00
1.12
70
55
AMPKα
1.00
0.74
1.00
0.39
35
25
Phos-S6
1.00
0.92
1.00
1.13
55
α-Tub

Fig. S1. mTORC1 signaling responds to different mechanical conditions

(A) Cell growth curve determined by CCK-8 assay at 0, 24, 48 and 72 h. Cell number of MDA-MB-231 was measured on stiff or soft substrates ($N_{stiff} = N_{soft} = 3$ independent samples, error bar: mean with SEM, ****$p < 0.0001$ by unpaired Student's *t* test).

(B) Representative images of overexpressed MLC-GFP in MDA-MB-231 cells. Scale bar, 20 μm.

(C) Cell growth curve determined by CCK-8 assay at 0, 24, 48 and 72 h under blebbistatin treatment. Cell number of MDA-MB-231 was measured in MLC- Rapamycin-, MLC+ Rapamycin-, MLC- Rapamycin+, MLC+ Rapamycin+ groups (error bar: mean with SEM, $ns_{\text{MLC- Rapamycin+ vs MLC- Rapamycin+}} > 0.9999$, ****$p < 0.0001$, **$p = 0.0036$ by one-way *ANOVA* for multiple comparison).

(D) Western blot showing Phos-S6 and total S6 levels in HRPE cells plated on stiff or soft substrates. α-tubulin is used as loading control.

(E) Western blot showing the Phos-S6 levels in stiff cultured cells treated with DMSO or ROCK inhibitor Y27632. α-tubulin is used as loading control.

(F) Western blot showing the Phos-S6 levels in soft cultured cells treated with DMSO or Rho activator II (Rho). α-tubulin is used as loading control.

(G) Western blot showing the mTOR, AMPKα and Phos-S6 levels in MDA-MB-231 and NIH3T3 cells cultured on stiff or soft substrates, with serum concentrations reducing to 1% during experiments. α-tubulin is used as loading control.

Figure S2

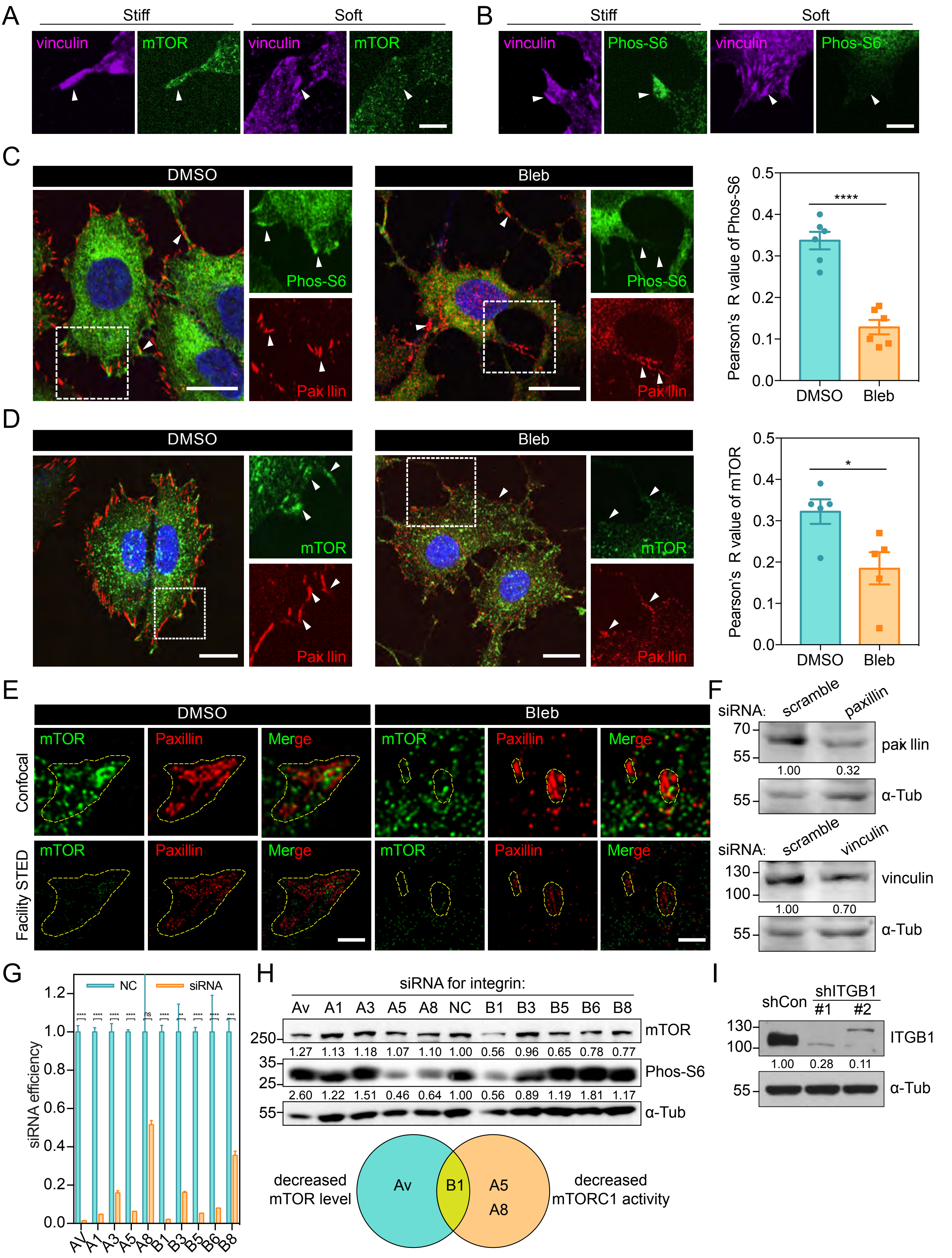
A
Stiff
Soft
vinculin
mTOR
B
Phos-S6
C
DMSO
Bleb
Paxillin
Pearson's R value of Phos-S6
****
D
Pearson's R value of mTOR
*
E
Confocal
Facility STED
Merge
F
siRNA:
scramble
paxillin
70
55
1.00
0.32
α-Tub
vinculin
130
100
0.70
G
NC
siRNA
siRNA efficiency
Av
A1
A3
A5
A8
B1
B3
B5
B6
B8
H
siRNA for integrin:
NC
250
35
25
1.27 1.13 1.18 1.07 1.10 1.00 0.56 0.96 0.65 0.78 0.77
2.60 1.22 1.51 0.46 0.64 1.00 0.56 0.89 1.19 1.81 1.17
decreased mTOR level
decreased mTORC1 activity
I
shCon
shITGB1
#1
#2
ITGB1
1.00
0.28
0.11

Fig. S2. Cell contractility induced mTORC1 activation is regulated by integrin-FAs

(A) Representative immunofluorescence images stained with mTOR (green) and vinculin (magenta) antibodies in MDA-MB-231 cells plated on stiff or soft substrates. Scale bar, 5 μm.

(B) Representative immunofluorescence images stained with Phos-S6 (green) and vinculin (magenta) antibodies in MDA-MB-231 cells plated on stiff or soft substrates. Scale bar, 5 μm.

(C) Left: representative immunofluorescence images stained with Phos-S6 (green) and paxillin (red) antibodies in MDA-MB-231 cells treated with DMSO or Bleb. Co-localization of Phos-S6 and paxillin is shown in enlarged boxes by white arrow heads. Scale bar, 20 μm. Right: Pearson's R value represents the overlap coefficient of Phos-S6 and paxillin ($N_{DMSO} = 6$, $N_{Bleb} = 6$, error bar: mean with SEM, ****$p < 0.0001$ by unpaired Student's *t* test).

(D) Left: representative immunofluorescence images stained with mTOR (green) and paxillin (red) antibodies in MDA-MB-231 cells treated with DMSO or Bleb. Co-localization of mTOR and paxillin is shown in enlarged boxes by white arrow heads. Scale bar, 20 μm. Right: Pearson's R value represents the overlap coefficient of mTOR and paxillin ($N_{DMSO} = 5$, $N_{Bleb} = 5$, error bar: mean with SEM, *$p = 0.0247$ by unpaired Student's *t* test).

(E) Representative images of confocal (top) and STED (bottom) showing the co-localization of mTOR (green) and paxillin (red) in MDA-MB-231 cells treated with DMSO or Bleb. Scale bar, 5 μm.

(F) Western blot showing the successful silencing of paxillin (top) and vinculin (bottom) in MDA-MB-231 cells using siRNA. α-tubulin is used as loading control.

(G) The knocked down efficiency of siRNA for integrin AV/A1/A3/A5/A8/B1/B3/B5/B6/B8 quantified by qPCR assay (error bar: mean with SEM, ****$p < 0.0001$, ***$p = 0.0010$, **$p = 0.0046$, ns = 0.4953 by unpaired Student's *t* test).

(H) Top: western blot showing the mTOR and Phos-S6 levels in siNC and integrin AV/A1/A3/A5/A8/B1/B3/B5/B6/B8 gene-silenced MDA-MB-231 cells. Bottom: Venn diagram showing the types of integrin that result in decreased mTOR protein levels and mTORC1 activity.

(I) Western blot showing the successful silencing of ITGB1 in MDA-MB-231 cells. α-tubulin is used as loading control.

Figure S3

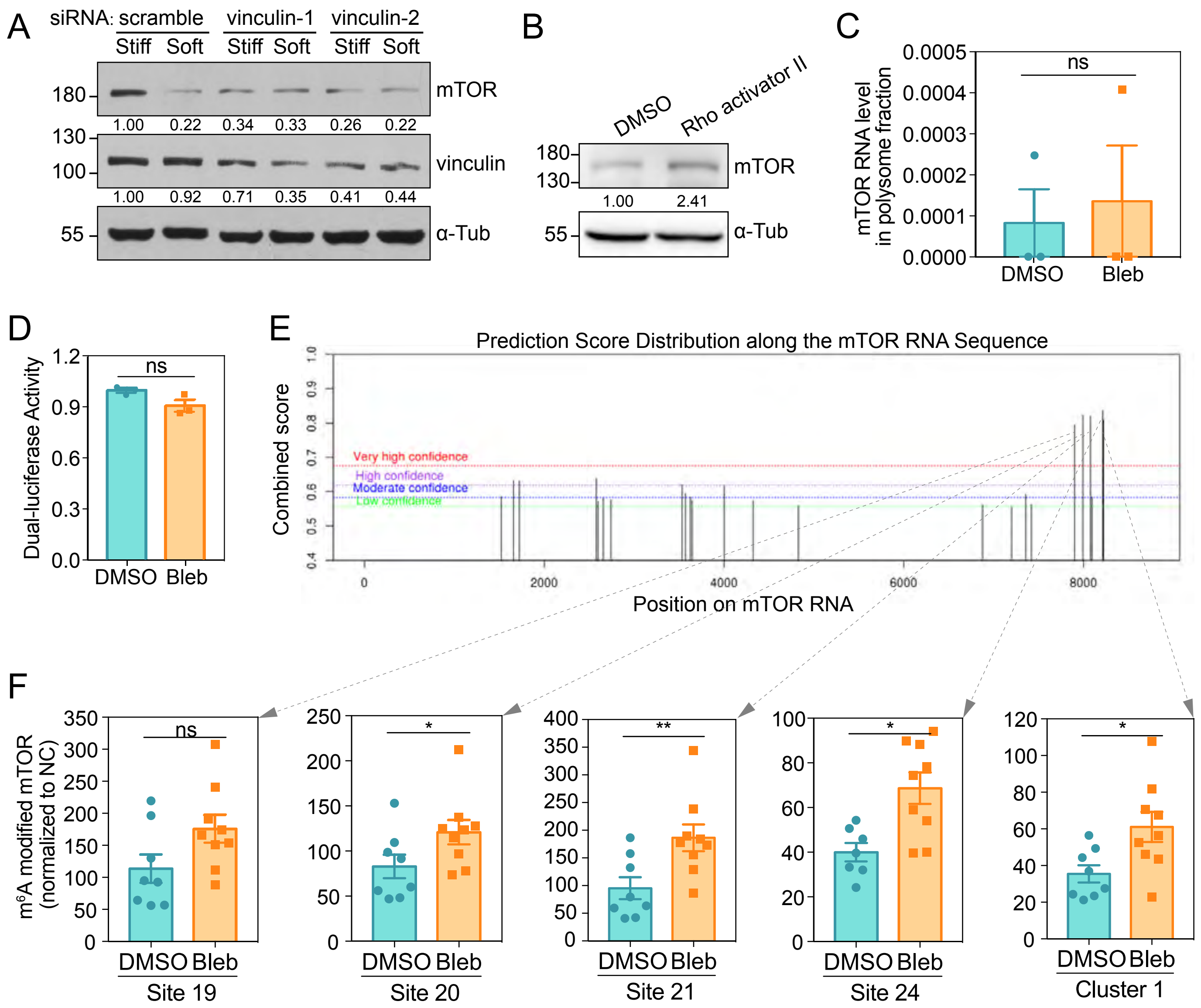
A
siRNA: scramble vinculin-1 vinculin-2
Stiff Soft Stiff Soft Stiff Soft
180
130
100
55
mTOR
1.00 0.22 0.34 0.33 0.26 0.22
vinculin
1.00 0.92 0.71 0.35 0.41 0.44
α-Tub
B
DMSO
Rho activator II
180
130
55
mTOR
1.00 2.41
α-Tub
C
mTOR RNA level in polysome fraction
ns
DMSO Bleb
D
Dual-luciferase Activity
ns
DMSO Bleb
E
Prediction Score Distribution along the mTOR RNA Sequence
Combined score
Very high confidence
High confidence
Moderate confidence
Low confidence
Position on mTOR RNA
F
m6A modified mTOR (normalized to NC)
ns
*
**
DMSO Bleb
Site 19
Site 20
Site 21
Site 24
Cluster 1

Fig. S3. m^6A modification of mTOR increases with reduced actomyosin contractility

(A) Western blot showing the mTOR and vinculin levels in scramble, si-vinculin-1, si-vinculin-2 MDA-MB-231 cells cultured on stiff or soft substrates. α-tubulin is used as loading control.

(B) Western blot showing the mTOR levels in cells treated with DMSO or Rho activator II. α-tubulin is used as loading control.

(C) The abundance of mTOR transcripts was measured by RT-qPCR in polysome fraction from the polysome profiling (error bar: mean with SEM, ns = 0.7542 by unpaired Student's *t* test).

(D) Dual-luciferase reporter assay showing transcriptional activity of mTOR mRNA in MDA-MB-231 cells treated with DMSO or Bleb ($N_{DMSO} = N_{Bleb} = 3$ independent experiments, error bar: mean with SEM, ns =0.0700 by unpaired Student's *t* test).

(E) Prediction by SRAMP showing the potential m^6A modification positions on mTOR mRNA sequence and its combined confidence score.

(F) The ratios of m^6A modified mTOR on site 19, site 20, site 21, site 24 and cluster 1 in DMSO and Bleb treatment groups ($N_{DMSO} = N_{Bleb} = 3$ independent experiments, error bar: mean with SEM, ns = 0.0564, $*p_{Site20} = 0.0267$, $**p_{Site\ 21} = 0.0038$, $*p_{Site24} = 0.0332$, $*p_{Cluster1} = 0.0176$ by paired Student's *t* test).

Figure S4

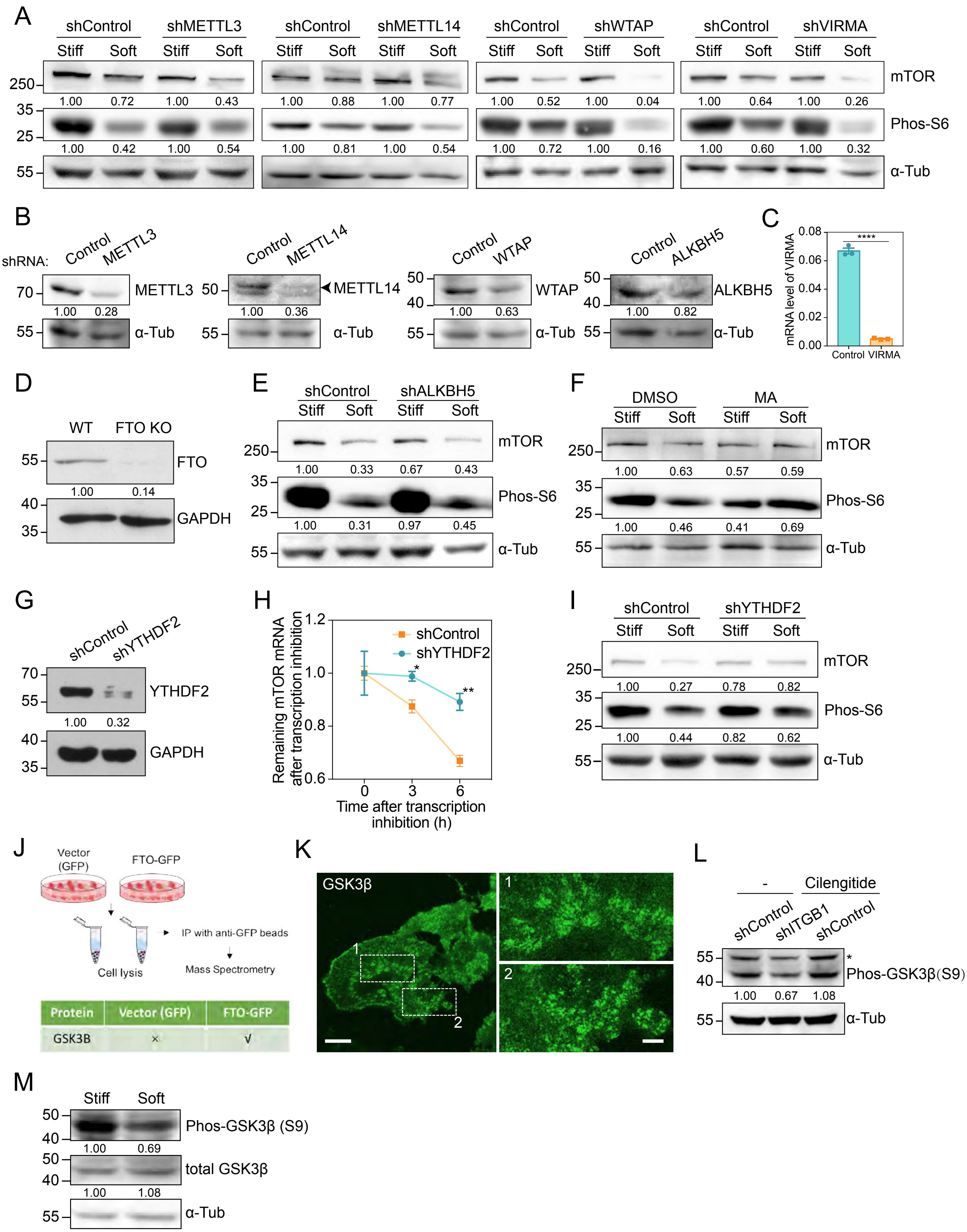
A
shControl
shMETTL3
shControl
shMETTL14
shControl
shWTAP
shControl
shVIRMA
Stiff Soft Stiff Soft
250
35
25
55
mTOR
Phos-S6
α-Tub
1.00 0.72 1.00 0.43
1.00 0.88 1.00 0.77
1.00 0.52 1.00 0.04
1.00 0.64 1.00 0.26
1.00 0.42 1.00 0.54
1.00 0.81 1.00 0.54
1.00 0.72 1.00 0.16
1.00 0.60 1.00 0.32
B
shRNA:
Control METTL3
70
METTL3
1.00 0.28
Control METTL14
50
METTL14
1.00 0.36
Control WTAP
50
40
WTAP
1.00 0.63
Control ALKBH5
ALKBH5
1.00 0.82
α-Tub
C
mRNA level of VIRMA
0.08
0.06
0.04
0.02
0.00
****
Control VIRMA
D
WT FTO KO
FTO
1.00 0.14
GAPDH
E
shControl shALKBH5
Stiff Soft Stiff Soft
mTOR
1.00 0.33 0.67 0.43
Phos-S6
1.00 0.31 0.97 0.45
α-Tub
F
DMSO MA
Stiff Soft Stiff Soft
mTOR
1.00 0.63 0.57 0.59
Phos-S6
1.00 0.46 0.41 0.69
α-Tub
G
shControl shYTHDF2
YTHDF2
1.00 0.32
GAPDH
H
Remaining mTOR mRNA after transcription inhibition
1.2
1.0
0.8
0.6
shControl
shYTHDF2
*
**
0 3 6
Time after transcription inhibition (h)
I
shControl shYTHDF2
Stiff Soft Stiff Soft
mTOR
1.00 0.27 0.78 0.82
Phos-S6
1.00 0.44 0.82 0.62
α-Tub
J
Vector (GFP)
FTO-GFP
IP with anti-GFP beads
Cell lysis
Mass Spectrometry
Protein | Vector (GFP) | FTO-GFP
GSK3B | × | √
K
GSK3β
1
2
L
-
Cilengitide
shControl shITGB1 shControl
*
Phos-GSK3β(S9)
1.00 0.67 1.08
α-Tub
M
Stiff Soft
Phos-GSK3β (S9)
1.00 0.69
total GSK3β
1.00 1.08
α-Tub

Fig. S4. The stability of mTOR transcripts is regulated by YTHDF2

(A) Western blot showing the mTOR and Phos-S6 levels in shControl, shMETTL3, shMETTL14, shWTAP and shVIRMA cells cultured on stiff or soft substrates. α-tubulin is used as loading control.

(B) Western blot showing the levels of METTL3/METTL14/WTAP/ALKH5 in shControl and corresponding shRNA-targeted MDA-MB-231 cells. α-tubulin is used as loading control.

(C) The knocked down efficiency of VIRMA quantified by qPCR assay (error bar: mean with SEM, ****$p < 0.0001$ by unpaired Student's *t* test).

(D) Western blot showing the levels of FTO in WT and FTO KO MDA-MB-231 cells. GAPDH is used as loading control.

(E) Western blot showing the levels of mTOR and Phos-S6 in shControl and shALKBH5 cells cultured on stiff or soft substrates. α-tubulin is used as loading control.

(F) Western blot showing the levels of mTOR and Phos-S6 in MDA-MB-231 cells supplemented with DMSO or meclofenamic acid (MA) on stiff or soft substrates. α-tubulin is used as loading control.

(G) Western blot showing the successful silencing of YTHDF2 in MDA-MB-231 cells. GAPDH is used as loading control.

(H) qPCR assay showing the remaining mTOR RNA level in shControl and shYTHDF2 cells after transcription inhibition using Actinomycin D on stiff or soft substrates ($N_{soft} = N_{stiff} = 3$ independent experiments, error bar: mean with SEM, *$p = 0.0206$, **$p = 0.0044$ by unpaired Student's *t* test).

(I) Western blot showing the mTOR and Phos-S6 levels in shControl and shYTHDF2 cells on stiff or soft substrates. α-tubulin is used as loading control.

(J) Schematic diagram of IP-MS detecting the interaction between GSK3β and FTO.

(K) Representative images of overexpressed GSK3β-AcGFP in MDA-MB-231 cells indicating its localization at the ventral side of cells. The dashed boxes are zoomed in on the right. Scale bar, 20 μm and 5 μm.

(L) Western blot showing the Phos-GSK3β (S9) levels in WT and shIGTB1 cells supplemented with Cilengitide. The asterisk indicates a nonspecific band. α-tubulin is used as loading control.

(M) Western blot showing phos-GSK3β and total GSK3β levels in MDA-MB-231 cells plated on stiff or soft substrates. α-tubulin is used as loading control.

Figure S5

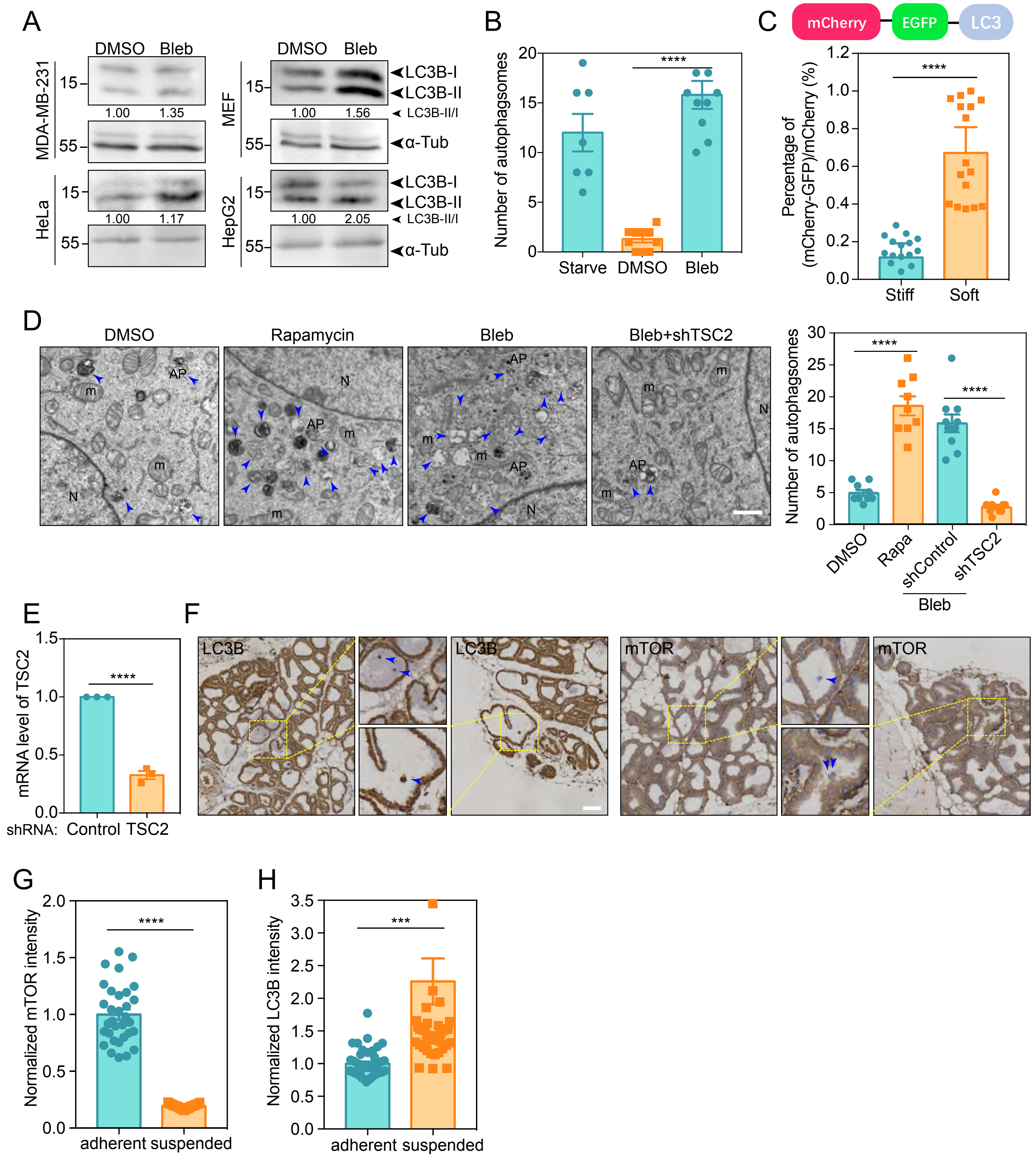

A
DMSO Bleb
MDA-MB-231
15
1.00 1.35
55
HeLa
1.00 1.17
MEF
LC3B-I
LC3B-II
1.00 1.56
LC3B-II/I
α-Tub
HepG2
1.00 2.05
B
Number of autophagsomes
****
Starve DMSO Bleb
C
mCherry EGFP LC3
Percentage of (mCherry-GFP)/mCherry (%)
****
Stiff Soft
D
DMSO Rapamycin Bleb Bleb+shTSC2
AP
m
N
Number of autophagsomes
DMSO Rapa shControl shTSC2
Bleb
E
mRNA level of TSC2
****
shRNA: Control TSC2
F
LC3B
mTOR
G
Normalized mTOR intensity
****
adherent suspended
H
Normalized LC3B intensity
***
adherent suspended

Fig. S5. ECM detachment hampers mTORC1 pathway and elevates autophagy in animal models

(A) Western blot showing the autophagy maker LC3B levels in MDA-MB-231, HeLa, MEF and HepG2 cells with or without Bleb treatment. α-tubulin is used as loading control.

(B) Quantification of autophagosomes number in TEM images of MDA-MB-231 cells under starve condition or treated with DMSO or Bleb (N_{starve} = 7, N_{DMSO} = 10, N_{Bleb} = 9, error bar: mean with SEM, ****$p < 0.0001$ by unpaired Student's *t* test).

(C) Top: schematic diagram of fluorescent tandem mCherry-EGFP-LC3 probe. Bottom: quantification of intensity of (mCherry–GFP) / mCherry on stiff and soft substrates (N_{stiff} = 15, N_{soft} = 16, error bar: mean with SEM, ****$p < 0.0001$ by unpaired Student's *t* test).

(D) Left: representative TEM images of MDA-MB-231 cells or shTSC2 cells treated with DMSO, Rapamycin, Bleb by chemical-fixation. The blue arrow heads indicate autophagosomes. The letter "N" stands for the nucleus and "m" stands for mitochondria. Scale bar, 1 μm. Right: quantification of autophagosomes number in images on the left (N_{DMSO} = $N_{Rapamycin}$ = N_{Bleb} = $N_{Bleb+shTSC2}$ = 9, error bar: mean with SEM, ****$p < 0.0001$ by unpaired Student's *t* test).

(E) The knocked down efficiency of TSC2 quantified by qPCR assay (error bar: mean with SEM, ****$p < 0.0001$ by unpaired Student's *t* test).

(F) Immunohistochemistry images showing the detection of mTOR and LC3B in lactating mammary glands sections from BALB/c mice. The yellow dashed boxes are zoomed in on the right. The blue arrow heads indicate ECM-detached cells. Scale bar, 100 μm.

(G) Quantification of mTOR fluorescence intensity in flank (adherent) and cavity (suspended) tumor cells in the nude mouse model of malignant ascites ($N_{adherent}$ = 32, $N_{suspended}$ = 43 cells, error bar: mean with SEM, ****$p < 0.0001$ by unpaired Student's *t* test).

(H) Quantification of LC3B fluorescence intensity in flank (adherent) and cavity (suspended) tumor cells in the nude mouse model of malignant ascites ($N_{adherent}$ = 43, $N_{suspended}$ = 46 cells, error bar: mean with SEM, ***$p = 0.0008$ by unpaired Student's *t* test).
